# Evaluation of Interleukin-8 (IL-8) Expression in Human Aortic Endothelial Cells (HAECs) and Antioxidant Activity of Isolated Compounds from the Endemic Hawaiian Plant *Pipturus albidus* (Māmaki)

**DOI:** 10.3390/molecules31193449

**Published:** 2026-09-28

**Authors:** Pornphimon Meesakul, Kyung Ah Kim, Yaru Si, Yutaka Kuroki, Aya Wada, Kaitlin Villafuerte, Zhenquan Jia, Ki Hyun Kim, Shugeng Cao

**Affiliations:** 1 Department of Pharmaceutical Sciences, Daniel K. Inouye College of Pharmacy, University of Hawai’i at Hilo, 200 W. Kawili St., Hilo, HI 96720, USA; pmeesak@hawaii.edu (P.M.); kaitlin.villafuerte@gmail.com (K.V.); 2 School of Pharmacy, Sungkyunkwan University, 2066 Seobu-ro, Jangan-gu, Suwon 16419, Republic of Korea; ruddk5480@g.skku.edu; 3 Department of Biology, University of North Carolina at Greensboro, 321 Mciver St. 312, Greensboro, NC 27412, USA; y_si@uncg.edu; 4 Delightex Pte. Ltd., 230 Victoria Street, #15-01/08 Bugis Junction Towers, Singapore 188024, Singapore; yutaka@delightexplorers.com (Y.K.); aya@delightexplorers.com (A.W.)

**Keywords:** *Pipturus albidus*, māmaki, phytochemistry, IL-8 expression, antioxidant, vitexin, structure–activity relationship

## Abstract

*Pipturus albidus* (commonly known as māmaki) is an endemic Hawaiian plant traditionally consumed as a medicinal herbal tea; however, its comprehensive potential for phytochemical composition and biological activities remains underexplored. In this study, we present the first comprehensive phytochemical characterization of the ethanol extract of *P. albidus*, identifying twelve compounds through chromatographic and spectroscopic analyses. The isolated compounds were subjected to a series of antioxidant assays, including 2,2-diphenyl-1-picrylhydrazyl (DPPH) radical scavenging, ferric reducing antioxidant power (FRAP), and oxygen radical absorbance capacity (ORAC). Cinchonain Ia (**2**), vitexin (**6**), and (7*S*,8*R*)-dihydrodehydro diconiferyl alcohol (**11**) demonstrated strong free radical-scavenging and reducing capacities. Evaluation of Interleukin-8 (IL-8) expression in TNF-α-stimulated human aortic endothelial cells (HAECs) revealed that catechin-(7,8-bc)-4a-(3,4-dihydroxyphenyl)-dihydro-2(3*H*)-pyranone (**3**) and *trans*-ferulic acid (**9**) significantly increased IL-8 mRNA expression (*p* < 0.05), indicating potential pro-inflammatory effects of these isolated constituents. In contrast, neither the crude extract (PE) nor vitexin (**6**) produced a statistically significant difference in IL-8 mRNA levels compared to the TNF-α control (*p* < 0.05). Quantitative LC-MS/MS analysis confirmed that caffeine, theobromine, theophylline, and EGCG were below the limit of detection in *P. albidus* leaf extracts, indicating that *P. albidus* possesses a phytochemical constituent distinct from that of green tea. In summary, *P. albidus* leaves contain unique phytochemicals with cell-free antioxidant activity, while cell-based assays indicate potential pro-inflammatory risks for compounds **3** and **9** and no significant anti-inflammatory suppression by PE or vitexin (**6**).

## 1. Introduction

*Pipturus albidus*—or māmaki (Hawaiian name)—is a small shrub endemic to the Hawaiian Islands that belongs to the Urticaceae family. It is a native Hawaiian plant with a well-documented history of traditional medicinal uses [1,2]. In Hawaiian ethnomedicine, several *Pipturus* species, including *P. albidus*, *P. forbesii*, *P. kauaiensis*, and *P. ruber*, are traditionally referred to as māmaki in Hawaiian, with *P. albidus* being the most widely recognized and utilized [1,2]. Traditionally, *P. albidus* leaves are steeped rather than boiled to prepare a healing herbal tea, which is consumed to alleviate stress and anxiety, treat allergies, and regulate blood pressure and cholesterol levels [2,3]. Beyond its leaves, multiple parts of this plant have been utilized, for example, the bark for making bark cloth (kapa), the roots as a natural dye source, and the fruits for nutritional purposes [3]. Recent scientific investigations have begun to characterize the nutritional and medicinal properties of *P. albidus*. Studies have revealed seasonal variations in macronutrients such as protein, dietary fiber, and minerals in both dried leaves and aqueous infusions [4,5]. Notably, *P. albidus* teas have been reported to contain higher concentrations of calcium, magnesium, and sodium than commercially available teas, including green and black teas [4]. Preliminary phytochemical screening has identified phenolic acids, including (+)-catechins, chlorogenic acid, and rutin, in *P. albidus* leaves, compounds recognized for their antioxidant and neuroprotective properties [6]. The total phenolic content and antioxidant capacity of *P. albidus* have been demonstrated to be comparable to or exceed those of other herbal teas, positioning it as a functional beverage with potential health benefits [6]. Beyond its nutritional profile, *P. albidus* exhibits promising bioactivity in various experimental models. Crude extracts have been shown to inhibit nuclear factor-kappa B (NF-κB) activation and nitric oxide production, suggesting anti-inflammatory potential, while ethanol-derived fractions demonstrated anti-cancer effects against breast cancer cells [7]. More recently, crude powders of *P. albidus* have been reported to reduce the accumulation of pathological proteins associated with dementia in mouse models of neurodegenerative diseases [8]. Additionally, methanolic extracts significantly modulate tyrosine hydroxylase (TH) and tryptophan hydroxylase (TPH) gene expression in PC-12 and Neuro-2A neuronal cell lines, indicating neuroactive potential relevant to mood regulation and neurotransmitter synthesis [9]. Despite these advances, the precise molecular mechanisms underlying the protective effects of *P. albidus* extracts remain unclear, and the specific bioactive compounds responsible for these activities have not been systematically isolated and characterized.

Oxidative stress, defined as an imbalance between reactive oxygen species (ROS) production and the capacity of antioxidant defenses, is a key driver in the pathogenesis of chronic diseases, including cardiovascular disease, neurodegenerative disorders, diabetes, and cancer [10,11]. In the vascular system, oxidative stress promotes endothelial dysfunction by upregulating the expression of pro-inflammatory cytokines. This pro-inflammatory activation enhances monocyte recruitment and initiates the formation of atherosclerotic lesions [12,13]. The tumor necrosis factor-alpha (TNF-α) signaling pathway is a key mediator of vascular inflammation, activating NF-κB and mitogen-activated protein kinases (MAPKs), which subsequently drive the transcription of pro-inflammatory genes [14]. Flavonoids, phenolic acids, and other plant-derived polyphenols have been widely investigated for their ability to scavenge relative oxygen species (ROS) and modulate inflammatory signaling pathways [15,16]. The structural diversity of these compounds underlies their varied bioactivities, with certain structural features, such as the number and position of hydroxyl groups, the presence of catechol or galloyl moieties, glycosylation patterns (*O*-glycosides versus *C*-glycosides), and the degree of conjugation, serving as key determinants of antioxidant and anti-inflammatory efficacy [17,18].

*P. albidus* tea contains a diverse range of constituents, including polyphenols, amino acids, and volatile compounds; however, the specific components responsible for its biological activity remain undefined, as systematic compound isolation and structure–activity relationship (SAR) analyses have not yet been conducted. Furthermore, methylxanthine compounds, including caffeine, theobromine, and theophylline, are widely present in common beverages and have been linked to disease-preventive effect mechanisms including adenosine receptor antagonism and phosphodiesterase inhibition [19,20]. Despite these recognized bioactivities, the methylxanthines content in *P. albidus* has not been characterized. Similarly, epigallocatechin gallate (EGCG), the major catechin in green tea with well-established antioxidant, neuroprotective, and anti-cancer properties [21], has not been quantified in *P. albidus*. Determining whether *P. albidus* contains EGCG or related catechins is essential in defining its phytochemical profile and distinguishing it from other tea beverages.

In this study, we provide the first comprehensive phytochemical investigation of a *P. albidus* ethanol extract, resulting in the isolation and structural elucidation of twelve distinct compounds. We systematically evaluated the antioxidant activities of purified constituents using three complementary assays including DPPH radical scavenging, ferric reducing antioxidant power (FRAP), and oxygen radical absorbance capacity (ORAC). Their inflammatory marker analysis was assessed in human aortic endothelial cells (HAECs) stimulated with TNF-α, focusing on IL-8 expression. In addition, targeted LC-MS/MS quantification was performed to determine the presence of key bioactive alkaloids (theobromine, theophylline, and caffeine) and EGCG. Collectively, these findings provide a detailed phytochemical characterization of *P. albidus* leaves and establish a biochemical rationale for further investigation of its potential therapeutic properties.

## 2. Results

### 2.1. Isolation

The crude extract of *P. albidus* leaves was systematically fractionated by liquid–liquid partition and subsequently purified using a combination of column chromatography and semi-preparative HPLC. This phytochemical investigation led to the isolation of twelve compounds (**1**–**12**), which were identified by spectroscopic analysis (^1^H NMR, ^13^C NMR, and mass spectrometry; Figures S1–S24) and comparison with literature data. The isolated compounds (Figure 1) were identified as catechin (**1**) [22], cinchonain Ia (**2**) [23], catechin-(7,8-bc)-4a-(3,4-dihydroxyphenyl)-dihydro-2(3*H*-pyranone (**3**) [24], catiguanin A (**4**) [25], (2*S*)-5,7,3′,5′-tetrahydroxyflavanone (**5**) [26], vitexin (**6**) [27], quercetin 3-rutinoside (**7**) [28], kaempferol 3-*O*-rutinoside (**8**) [29], *trans*-ferulic acid (**9**) [30], 5-*O*-caffeoylquinic acid (**10**) [31], (7*S*,8*R*)-dihydrodehydro diconiferyl alcohol (**11**) [32], and 4-hydroxybenzoic acid (**12**) [33]. These compounds represent diverse phytochemical classes, including catechin derivatives, flavonoids (flavones and flavanones), phenylpropanoids, hydroxycinnamic acids, and benzoic acids.

### 2.2. Evaluation of IL-8 Expression in HAECs

To evaluate whether *P. albidus* extract (PE) or its isolated constituents alter vascular inflammatory signaling, their effects on TNF-α-induced IL-8 mRNA expression were evaluated in HAECs. Quantitative real-time PCR (qPCR) confirmed robust induction of IL-8 mRNA following TNF-α stimulation. Due to limited isolation yields for several pure constituents, cell-based evaluation was focused on the crude extract (PE) and prioritized isolated compounds (**3**, **6**, and **9**).

As shown in Figure 2, treatment with crude PE or vitexin (**6**) resulted in no statistically significant difference in TNF-α-induced IL-8 mRNA levels relative to the TNF-α control group (*p* < 0.05), demonstrating only minor, non-significant numerical variations. In contrast, treatment with compound **3** [catechin-(7,8-bc)-4a-(3,4-dihydroxyphenyl)-dihydro-2(3*H*)-pyranone] and compound **9** (*trans*-ferulic acid) led to a statistically significant increase in IL-8 mRNA expression compared to the TNF-α control (*p* < 0.05), indicating a pro-inflammatory effect under these cellular testing conditions.

### 2.3. Antioxidant Assay

The antioxidant potential of the *P. albidus* ethanol extract (PE) and its pure isolates was systematically assessed through a range of chemical and biological assays: 2,2-diphenyl-1-picrylhydrazyl (DPPH) radical scavenging (Figure 3), Ferric Reducing Antioxidant Power (FRAP) (Figure 4), and Oxygen Radical Absorbance Capacity (ORAC) (Figure 5).

#### 2.3.1. DPPH Free Radical-Scavenging Activity

The crude PE displayed significant total antioxidant capacity (Figure 3), comparable to that of synthetic antioxidant Trolox. Among the isolated compounds (Figure 1), cinchonain Ia (**2**) and vitexin (**6**) demonstrated the strongest radical-scavenging activity, significantly exceeding that of PE. Compound (**11**) also demonstrated significantly higher activity than PE, whereas compound (**3**), as well as compounds **4**, **5**, **7**, **8**, **9**, **10**, and **12**, demonstrated significantly lower DPPH radical-scavenging activity compared to PE (*p* < 0.05) (Figure 3). Only compound **1** showed no statistically significant difference relative to PE (*p* < 0.05) (Figure 3).

#### 2.3.2. Ferric Reducing Antioxidant Power (FRAP) Assay

Among the isolated constituents, vitexin (**6**) exhibited the highest reducing power, significantly exceeding the activity of PE. Catiguanin A (**4**) and (7*S*,8*R*)-dihydrodehydro diconiferyl alcohol (**11**) also demonstrated significantly reduced capacity, suggesting that their structural features favor efficient electron donation. In contrast, all other isolated constituents, including cinchonain Ia (**2**), catechin-(8,7-e)-4β-(3,4-dihydroxyphenyl)-dihydro-2(3*H*)-pyranone (**3**), and *trans*-ferulic acid (**9**), as well as compounds **1**, **5**, **7**, **8**, **10**, and **12**, exhibited significantly lower ferric reducing power compared to PE (*p* < 0.05) (Figure 4).

#### 2.3.3. Oxygen Radical Absorbance Capacity (ORAC) Assay

The ethanol extract of *P. albidus* (PE) demonstrated moderate peroxyl radical-scavenging activity (Figure 5), indicating its potential to protect vascular membranes from oxidative degradation. Cinchonain Ia (**2**) and (7*S*,8*R*)-dihydrodehydro diconiferyl alcohol (**11**) exhibited the highest peroxyl radical-scavenging activity—significantly greater than that of crude PE. Catechin-(8,7-*e*)-4β-(3,4-dihydroxyphenyl)-dihydro-2(3*H*)-pyranone (**3**), catiguanin A (**4**), and vitexin (**6**) also demonstrated significantly higher activity than PE. In contrast, all remaining compounds (**1**, **5**, **7**, **8**, **9**, **10**, and **12**) showed significantly lower lipid radical-scavenging activity relative to PE, with compound **12** displaying the weakest activity (Figure 5).

### 2.4. Quantification in P. albidus and Green Tea Extracts

Quantification of four *P. albidus* (māmaki) extracts was performed alongside a commercially available green tea extract, which served as a positive control to validate the performance of the LC–MS/MS method. The concentrations of theobromine, theophylline, caffeine, and EGCG are summarized in Table S2. All *P. albidus* extracts showed analyte levels below the limit of detection (ND, not detected), indicating that these bioactive compounds are either absent or present only in trace amounts below the analytical sensitivity of the method. In contrast, the green tea extract contained measurable levels of all analytes, including caffeine at 5.495%, EGCG at 3.496%, theobromine at 0.044%, and theophylline at 0.009%. The consistency and magnitude of these values in the positive control confirm the accuracy, sensitivity, and reliability of the analytical method, thereby supporting the validity of non-detectable results obtained for the *P. albidus* extracts.

The absence of detectable methylxanthines and catechins in *P. albidus* leaves is a noteworthy finding. Unlike *Camellia sinensis* and other tea-producing species, which characteristically contain these common bioactive constituents, *P. albidus* displays a distinctly phytochemical profile. This contrast suggests that the physiology or pharmacological effects attributed to *P. albidus* are unlikely to be mediated by theophylline, theobromine, caffeine, or EGCG at the concentrations present in the leaf extracts. Future investigations should focus on identifying alternative bioactive constituents in *P. albidus* that may underlie its reported biological activities.

In summary, the quantitative analysis demonstrated that *P. albidus* leaves contain negligible or undetectable levels of theophylline, theobromine, EGCG, and caffeine, in sharp contrast to the green tea control (Table S2). The consistent results and robust analyte recovery observed in the positive control confirm the reliability and sensitivity of the LC–MS/MS analytical method. These findings highlight the distinct phytochemical profile of *P. albidus* and suggest that its biological activities may be driven by as-yet-unidentified secondary metabolites. Further investigation of these unique constituents may provide valuable insights into the bioactive potential and therapeutic applications of *P. albidus*.

## 3. Discussion

This study presents the first comprehensive phytochemical constituents from an ethanol extract of endemic Hawaiian plant *P. albidus* (Māmaki). Twelve compounds (Figure 1) were successfully identified through chromatographic and spectroscopic analyses, including catechin derivatives (**1**–**4**), flavonoids (**5**–**8**), phenylpropanoid (**11**), cinnamic acid, and benzoic acid derivatives (**9**, **11**, and **12**). Importantly, targeted quantitative analysis showed that methylxanthines (caffeine, theobromine, and theophylline) and major green tea catechin EGCG were below the limits of detection in the analyzed samples, supporting the characterization of *P. albidus* as a caffeine-free botanical with a phytochemical profile distinct from *C. sinensis*. Systematic evaluation of isolated pure compounds using complementary antioxidant assays (DPPH, FRAP, and ORAC) alongside inflammatory response assessment via IL-8 expression in TNF-α-stimulated HAECs revealed pronounced variations in bioactivity. IL-8 is a potent cytokine that plays a critical role in recruiting monocytes and neutrophils to sites of endothelial injury [34]. Among all tested constituents, vitexin (**6**) consistently emerges as the most potent compound across all assays, demonstrating strong radical-scavenging capacity, high reducing power, and significant suppression of TNF-α-induced IL-8 expression. At 10 μg/mL, the crude PE showed a significant capacity to scavenge DPPH radicals (Figure 3) and exhibited strong ferric-reducing capacity in the FRAP assay (Figure 4). Furthermore, the crude extract (PE) effectively scavenged hydrogen peroxide in a dose-dependent manner, while its isolated pure constituents demonstrated significant radical-scavenging and reducing capacities compared to the crude extract at their evaluated single concentrations (Figure 3, Figure 4 and Figure 5). Collectively, these results highlight the broad-spectrum antioxidant potential of *P. albidus*.

Crucially, our targeted quantitative analysis systematically evaluated the presence of key bioactive alkaloids and polyphenols commonly found in commercial teas: theophylline, theobromine, caffeine, and epigallocatechin gallate (EGCG). In striking contrast to green tea, which contained measurable levels of all four analytes (caffeine at 5.495% and EGCG at 3.496%), all four *P. albidus* extracts exhibited concentrations below the limit of detection (ND) for these compounds. This confirmed the absence of detectable methylxanthines and catechins, highlighting a distinct phytochemical profile. These findings suggest that the functional properties attributed to *P. albidus* are driven by its unique blend of identified phenolic compounds, including catechin derivatives and cinnamic acid-based molecules rather than the common tea-associated compounds found *C. sinensis* (green tea).

A central finding of this study is the marked variation in bioactivity among the isolated pure compounds. While some constituents exhibited measurable antioxidant potential, vitexin (**6**) emerged as a particularly potent modulator of both ROS and pro-inflammatory signaling. In contrast, catechin-(8,7-*e*)-4β-(3,4-dihydroxyphenyl)-dihydro-2(3*H*)-pyranone (**3**) and *trans*-ferulic acid (**9**) demonstrated significantly lower efficacy across these functional endpoints. These results demonstrate a clear distinction with respect to the biological potency of these compounds, particularly the compounds’ ability to scavenge ROS and suppress TNF-α-induced IL-8 in HAECs (Figure 2). This differential activity underscores the importance of structural diversity within the *P. albidus* phytochemical constituents and highlights vitexin (**6**) as a key contributor to its antioxidant and pro-inflammatory effects.

### 3.1. Structure–Activity Relationships (SAR): Understanding the Molecular Basis of Bioactivity

#### 3.1.1. Vitexin (**6**): Superior Multi-Mechanism Antioxidant and Inflammatory Response Activity

Vitexin (**6**) was the most consistently effective constituent across all antioxidant and inflammatory response assays, firmly establishing it as the principle bioactive compound in *P. albidus*. This compound exhibited strong activity across all cell-free antioxidant assays (DPPH, FRAP, and ORAC), significantly exceeding crude PE performance. Structurally, vitexin (**6**) is a *C*-glycosyl flavone in which an apigenin aglycone is linked to a glucose moiety through a carbon–carbon bond at the C-8 position.

Its exceptional performance is fundamentally linked to its unique *C*-glycosyl structure. In most flavonoids, sugar moieties are linked to the aglycone via an *O*-glycosidic bond, which is sensible to hydrolysis by intestinal and systemic enzymes [18]. This structural feature is central to vitexin’s enhanced bioactivity for several reasons.

First, the *C*-*C* bond in vitexin is remarkably stable compared to the *O*-glycosidic bonds found in most dietary flavonoids, ensuring that the intact molecule can reach the vascular endothelium in bioactive concentrations [35]. *O*-glycosides are rapidly hydrolyzed, releasing the aglycone, which often displays reduce bioavailability or undergoes accelerated metabolism [35,36]. This enhanced stability translates to sustained antioxidant activity in vivo and explains vitexin (**6**)’s superior performance in the ORAC assay, which evaluates antioxidant capacity over an extended reaction period.

Second, vitexin (**6**) contains key hydroxyl groups at the 4’ position of the B-ring, as well as the 5 and 7 positions of the A-ring, which serve as highly effective sites for hydrogen atom transfer (HAT) and single electron transfer (SET) during reactive oxygen species (ROS) neutralization [17,18,37]. Evidence indicates that the 4’-OH group is especially important because the resulting phenoxyl radical is stabilized by the resonance delocalization across the conjugated flavone backbone [35]. Quantum-chemical calculations and SAR studies have demonstrated that the presence of hydroxyl groups at both the 4’ and 7 positions significantly enhances radical-scavenging capacity [38,39]. This explains vitexin (**6**)’s exceptional DPPH-scavenging activity and FRAP-reducing power, both of which depend on efficient electron or hydrogen donation.

Third, in TNF-α-stimulated HAECs, vitexin (**6**) produced a numerical reduction in IL-8 mRNA levels compared to the TNF-α control group (Figure 2), while previously published literature reports indicate that a major mechanism of action of vitexin (**6**) in endothelial cells involves the inhibition of APEX1, a protein that plays a dual role in DNA repair and the regulation of cellular redox signaling [40]. Zhao and colleagues demonstrated that suppression prevents the downstream activation of the NF-κB p65 subunit, thereby preventing the transcription of pro-inflammatory chemokines [40]. While we did not directly measure APEX1 or downstream phosphorylation in this study, this reported pathway provides a plausible framework for the transcriptional suppression of IL-8 observed in our qPCR assays. In addition, vitexin (**6**) has been reported to modulates the p38 MAPK and ERK signaling pathways, both of which are central mediators of cytokine-induced endothelial dysfunction [41]. While we did not directly measure these intracellular signaling pathways in our current study, our transcriptional finding that vitexin (**6**) reduced the trend of IL-8 mRNA expression aligns with these established literature-reported mechanisms and warrants future Western blot verification in HAECs.

#### 3.1.2. Catiguanin A (**4**) Diconiferyl Alcohol or Phenylpropanoid (**11**): Selective Activity Profiles

Catiguanin A (**4**) and (7*S*,8*R*)-dihydrodehydro diconiferyl alcohol (**11**) both exhibited significantly higher activity than the crude extract in FRAP and ORAC assays, whereas their DPPH activity was more modest. This selective activity profile can be explained by their unique structural characteristics. Catiguanin A (**4**) is a catechin–phenylpropanoid conjugate, formed by the linkage of a catechin unit with a dihydrocaffeic acid (DHCA) moiety. The numerous phenolic hydroxyl groups present on both the catechin and DHCA moieties provide abundant sites for quenching peroxyl radicals, explaining the strong ORAC response. However, the bulky structure of catiguanin A (**4**) may introduce steric hindrance, limiting its interaction with the small, planar DPPH radical and resulting in weaker DPPH scavenging [25,42].

In contrast, dihydrodehydro diconiferyl alcohol (**11**) is a lignan composed of two dihydroconiferyl alcohol units. Lignans are known for their antioxidant potential due to the presence of methoxy and hydroxyl groups on aromatic rings [32,43]. The flexible conformation of **11** allows it to adopt geometries favorable for interactions with various radical species, supporting its strong performance across all three assays. Compound **11** exhibited significantly higher activity than the crude extract (PE) in FRAP and ORAC assays, but its DPPH activity was more modest, positioning it as a secondary lead compound following vitexin (**6**). This selective activity pattern can be explained by its unique structural features.

#### 3.1.3. Compounds with Reduced Activity: Structural Limitations

Catechin-(8,7-*e*)-4*β*-(3,4-dihydroxyphenyl)-dihydro-2(3*H*)-pyranone (**3**) exhibited significantly lower activity than PE across all antioxidant assays (DPPH, FRAP, and ORAC) and, notably, increased IL-8 expression in HAECs. Structurally, compound **3** is a modified catechin featuring a fused pyranone ring at the 7 and 8 positions, linked to an additional 3,4-dihydroxyphenyl group. This (7,8-bc) ring fusion introduces conformational rigidity to the molecule, limiting the molecular flexibility typically required for optimal interactions with radical species and biological targets.

The conformational constraint imposed by the pyranone ring likely hinders the molecule’s ability to adopt geometries that maximize overlap with radical species, thereby reducing its antioxidant efficacy [24]. More importantly, this rigidity may sterically block productive interactions with key signaling proteins, which require specific binding pocket geometries for inhibition. Prior work by Shamsudin and colleagues [44] demonstrated that structural modifications at the C-3 or C-7 positions of flavonoids can markedly reduce inflammation despite maintaining antioxidant properties. Although catechins are generally potent inhibitors of IL-1β and NF-κB, substitution at the C-3 or C-7 positions have been shown to diminish anti-inflammatory activity, even if they enhance other properties, such as antidiabetic activity [18]. This divergence suggests that, while compound **3** retains some antioxidant potential, its ability to modulate the specific signaling pathways controlling IL-8 in HAECs is compromised relative to the more flexible and targeted flavonoid vitexin (**6**). The observed increased IL-8 expression further implies that compound **3** may act as a partial agonist or modulator of pro-inflammatory pathways.

*Trans*-ferulic acid (**9**) also exhibited significantly lower antioxidant activity and increased IL-8 expression. Ferulic acid (4-hydroxy-3-methoxycinnamic acid) is a hydroxycinnamic acid containing a single phenolic hydroxyl group and a methoxy substituent, features that limit its electron-donating capacity. While ferulic acid is generally recognized as an antioxidant, its activity is highly concentration-dependent and context-dependent [45,46]. One explanation for the opposing effects of ferulic acid is its potential to act as a pro-oxidant in environments with high basal oxidative stress in the presence of transition metals (Fe^3+^ and Cu^2+^) or peroxidases, which may engage in deleterious secondary reactions if not efficiently quenched by endogenous antioxidants [45]. TNF-α-stimulated endothelial cells exhibit elevated ROS production and an oxidatively stressed microenvironment, which may favor ferulic acid’s pro-oxidant behavior. Additionally, ferulic acid has been reported to exhibit weaker inhibition of NF-κB than larger polyphenols, such as flavonoids [47], consistent with our observation of increased IL-8 expression.

Finally, 4-hydroxybenzoic acid (**12**) exhibited the weakest antioxidant activity among tested compounds. As the simplest phenolic compound in this study, it contains only a single hydroxyl group and lacks extended conjugation or additional electron-donating substituents. The resulting phenoxy radical is poorly stabilized, severely limiting its antioxidant capacity for hydrogen atom transfer or electron donation and explaining its minimal activity across all assays [33,48].

#### 3.1.4. Pro-Inflammatory Effects and Toxicological Considerations of Compounds **3** and **9**

Rather than demonstrating anti-inflammatory protection, treatment with catechin-(7,8-bc)-4a-(3,4-dihydroxyphenyl)-dihydro-2(3*H*)-pyranone (**3**) and *trans*-ferulic acid (**9**) significantly increased IL-8 mRNA expression in TNF-α-stimulated HAECs (*p* < 0.05). These findings highlight potential pro-inflammatory activity and toxicological concerns associated with specific isolated constituents of *P. albidus*. The structural rigidity of the pyranone-fused ring in compound **3** or potential pro-oxidant secondary reactions of *trans*-ferulic acid (**9**) under oxidative stress conditions may account for this cytokine upregulation. From a safety perspective, these results indicate that high-dose exposure or enriched supplementation with isolated compounds **3** or **9** could exacerbate vascular endothelial inflammation, posing potential safety risks for vulnerable populations with pre-existing inflammatory or cardiovascular disorders.

### 3.2. Assay-Specific Activity Patterns: Mechanistic Insights

The differential activity of certain compounds across assays provides valuable mechanistic insights. For instance, cinchonain Ia (**2**) displayed strong DPPH-scavenging and ORAC capacity but only weak FRAP activity, suggesting that compound **2** is particularly effective in HAT-mediated radical scavenging but less efficient in SET-mediated electron donation. Structurally, cinchonain Ia (**2**) contains multiple hydroxy groups, which enhance hydrogen-donating capacity but contribute less effectively to electron donation [23].

In contrast, *O*-glycosylated flavonoids such as quercetin 3-rutinoside (**7**) and kaempferol 3-*O*-rutinoside (**8**) showed significantly lower ORAC activity to PE but completely low DPPH and FRAP activity. The ORAC assay measures sustained antioxidant capacity during prolonged incubation over time, and the susceptibility of *O*-glycosidic bonds to hydrolysis becomes a critical factor, leading to the gradual loss of sugar moiety and altered antioxidant properties [49,50]. This underscores the advantage of *C*-glycosides such as vitexin (**6**), which contains a *C*-*C* glycosidic linkage that remains intact throughout the assay, preserving both structural integrity and functional activities.

### 3.3. Phytochemical Profile and Biochemical Profile: Distinguishing P. albidus from Green Tea

Our quantitative LC-MS/MS analysis confirmed that *P. albidus* contains no detectable levels of caffeine, theobromine, theophylline, or EGCG. In contrast, green tea exhibited substantial levels of these compounds, containing caffeine (5.495%), EGCG (3.496%), theobromine (0.044%), and theophylline (0.009%). This finding has important implications for both the phytochemical classification of *P. albidus* and its potential health applications.

The absence of detectable caffeine positions *P. albidus* as a naturally caffeine-free herbal option that may serve as a suitable alternative for individuals seeking to limit or avoid dietary stimulants. Although EGCG is widely recognized for its beneficial properties, in literature, high doses have been linked to potential adverse effects, including hepatotoxicity and drug interactions [51,52]. While our analytical data confirmed that EGCG was below the limit of detection in the analyzed *P. albidus* samples, distinguishing its phytochemical profile from that of *C. sinensis*, this chemical difference does not establish the overall clinical safety of *P. albidus*. A comprehensive determination of the safety of *P. albidus* preparations requires detailed future toxicological evaluations to assess its full chemical matrix, dose-response profiles, metabolic fate, and potential compound–medication interactions.

Instead, the biological effects of *P. albidus* appear to be driven by its unique profile of specialized metabolites, including catechin derivatives (particularly cinchonain Ia (**2**) and catiguanin A (**4**)), *C*-glycosyl flavonoids (vitexin (**6**)), and lignans ((7*S*,8*R*)-dihydrodehydro diconiferyl alcohol (**11**)). This unique phytochemical signature not only differentiates *P. albidus* from green tea but also indicates that its health-promoting properties are mediated through mechanisms independent of adenosine receptor antagonism or EGCG-related pathways.

The traditional medicinal preparations of *P. albidus* leaves used in Hawaiian ethnomedicine to alleviate stress, regulate blood pressure, and treat allergies serve as a useful historical context for our study. This context is important, i.e., that it is well established that oxidative stress and chronic low-grade inflammation are well-recognized contributors to hypertension, endothelial dysfunction, and allergic responses [53,54]. While our in vitro chemical assays demonstrated strong antioxidant profiles for vitexin (**6**), catiguanin A (**4**), and (7*S*,8*R*)-dihydrodehydro diconiferyl alcohol (**11**), these chemical observations cannot be directly extrapolated to systemic in vivo efficacy. Future pharmacokinetic and clinical studies are required to determine if these compounds reach active physiological concentrations in human tissues following the consumption of *P. albidus* (māmaki) tea.

Moreover, the ability of vitexin (**6**) to suppress TNF-α-induced IL-8 expression in endothelial cells in vitro provides a preliminary biochemical rationale for investigating its potential role in cardiovascular health. IL-8 is an established chemokine that contributes to monocyte recruitment during early vascular inflammation [55]. While our study was limited to transcriptional qPCR screening and did not directly measure monocyte adhesion, cell infiltration, adhesion molecule expression, or physical vascular function, the down-regulation of IL-8 transcripts by vitexin (**6**) suggests a plausible protective pathway that warrants further functional evaluation. Although separate literature reports show that vitexin (**6**) protects against flow-induced endothelial dysfunction in mice [40] and exhibits cardio-protection in myocardial ischemia–reperfusion models [56], direct functional cell-adhesion assays and animal model studies are required to confirm whether our observed transcriptional changes translate into a physical reduction in monocyte recruitment or attenuated plaque formation in vivo. This suggests that, by inhibiting IL-8 transcripts in vitro, vitexin (**6**) may reduce monocyte recruitment, a hypothesis that requires direct validation in functional cell-adhesion and in vivo animal assays.

Future research should prioritize in vivo pharmacokinetics and the bioavailability of vitexin (**6**) and other bioactive compounds in *P. albidus*. Clinical trials evaluating the effects of *P. albidus* tea consumption on markers of oxidative stress, inflammation, and cardiovascular function would provide valuable evidence for its therapeutic potential. Additionally, the isolation and standardization of vitexin (**6**)-enriched extracts could lead to the creation and development of nutraceutical formulations with targeted vascular protective properties.

## 4. Materials and Methods

### 4.1. General Experimental Procedures

Optical rotations were measured using a Jasco P-2000 polarimeter (Jasco, Easton, MD, USA). Ultraviolet (UV) spectra were recorded on an Agilent 8453 UV−visible spectrophotometer (Agilent Technologies, Santa Clara, CA, USA). Nuclear magnetic resonance (NMR) spectra were obtained on a Bruker AVANCE III HD 850 NMR spectrometer (Bruker, Karlsruhe, Germany) equipped with a 5 mm TCI CryoProbe operating at 850 MHz for ^1^H, with chemical shifts reported in parts per million (ppm, *δ*). Semi-preparative HPLC was performed on a Shimadzu Prominence HPLC System equipped with SPD-20A/20AV Series Prominence HPLC UV-Visible detectors (Shimadzu, Tokyo, Japan) and a Phenomenex Luna C_18_ column (250 × 10 mm, 5 μm; flow rate: 2 mL/min; Phenomenex, Torrance, CA, USA). Liquid chromatography–mass spectrometry (LC/MS) analyses were conducted using an Agilent 1200 Series HPLC system with a diode array detector and a 6130 Series ESI mass spectrometer, employing an analytical Kinetex C_18_ 100 Å column (100 × 2.1 mm, 5 μm; flow rate: 0.3 mL/min; Phenomenex, Torrance, CA, USA). All high-resolution electrospray ionization mass spectrometry (HR-ESI-MS) data were acquired on an Agilent 6545 Q-TOF LC/MS spectrometer (Agilent Technologies, Santa Clara, CA, USA). RP-C_18_ silica gel (Merck, Rahway, NJ, USA, 230–400 mesh) and Silica gel 60 (230−400 mesh; Merck) were used for column chromatography. Sephadex LH-20 (Pharmacia, Uppsala, Sweden) and Diaion^®^ HP-20 (Supelco, Bellefonte, PA, USA) were employed for molecular sieve and reverse-phase column chromatographic separations. Thin-layer chromatography (TLC) was performed on Merck precoated silica gel F_254_ plates and RP-C_18_ F_254_s plates, with visualization under UV light or by heating after spraying with anisaldehyde–sulfuric acid reagent.

### 4.2. Plant Material, Extraction, and Isolation

Leaves of *P. albidus* were collected in April 2020 at Hawaii Forest Farm (HFF) by the farm owner (Grant Ferrier) and co-workers. The leaves were shade-dried and milled into fine powder. The resulting material (about 4.55 kg) was extracted twice with 50 L of 100% ethanol each time by maceration. The combined extracts were filtered and concentrated under reduced pressure to yield approximately 455.0 g of crude extract. The resulting crude extract was subsequently solvent-partitioned with hexane, dichloromethane, ethyl acetate, and *n*-butanol, affording the following fractions: hexane (27.2 g), dichloromethane (5.5 g), ethyl acetate (5.3 g), and *n*-butanol (26.4 g).

The hexane fraction (27.2 g) was subjected to column chromatography (CC) on HP-20 using a stepwise gradient of H_2_O, MeOH, and acetone, yielding a crude methanol fraction (10.7 g). This was further separated by CC on silica gel with a gradient of CH_2_Cl_2_/MeOH (50:1 to 100% MeOH), affording seven subfractions (HA–HG). Fraction HF (336.1 mg) was then further purified by CC on Sephadex LH-20 with H_2_O/MeOH (1:1 to 100% MeOH), yielding four subfractions (HF1–HF4). Finally, subfraction HF3 (55.0 mg) was purified by semi-preparative reversed-phase HPLC (Phenomenex Luna C_18_, 250 × 10.0 mm i.d., 10 µm) under isocratic conditions with 45% MeOH/H_2_O at a flow rate of 2 mL/min, yielding compounds **7** (1.6 mg) and **8** (1.4 mg).

The ethyl acetate fraction (5.3 g) was separated by column chromatography (CC) on silica gel using a gradient of CH_2_Cl_2_/MeOH (30:1 to 100% MeOH), yielding seven fractions (EA–EG). Fraction EB (341.8 mg) was further purified by CC on Sephadex LH-20 (100% MeOH), affording five subfractions (EB1–EB5). Subfraction EB5 was obtained as pure compound **5** (5.7 mg). Subfraction EB3 (35.4 mg) was purified by semi-preparative reversed-phase HPLC under isocratic conditions with 43% MeOH/H_2_O (flow rate: 2 mL/min), yielding compound **9** (1.0 mg). Fraction EC (100.4 mg) was subjected to CC on Sephadex LH-20 (100% MeOH), resulting in four subfractions (EC1–EC4). Compound **11** (1.5 mg) was obtained from subfraction EC2 (8.2 mg) by semi-preparative reversed-phase HPLC using 39% MeOH/H_2_O. Compounds **10** (1.6 mg) and **12** (1.6 mg) were purified from subfraction EC3 (17.2 mg) using semi-preparative reversed-phase HPLC with 40% MeOH/H_2_O. Fraction ED (1.0 g) was further fractionated by CC on RP-C18 using a gradient solvent system of MeOH/H_2_O (20–100% MeOH), affording four subfractions (ED1–ED4). Subfraction ED2 (339.4 mg) was then subjected to CC on silica gel with a gradient of CH_2_Cl_2_/MeOH (10:1 to 100% MeOH), yielding six subfractions (ED2A–ED2F). Purification of subfraction ED2B (124.5 mg) by semi-preparative reversed-phase HPLC with 39% MeOH/H_2_O provided compounds **1** (4.7 mg), **2** (2.0 mg), **3** (2.0 mg), and **4** (1.9 mg). Compound **6** (0.2 mg) was purified from subfraction ED2D (39.0 mg) by semi-preparative reversed-phase HPLC using 35% MeOH/H_2_O.

Dry powder of *P. albidus* leaves (four types), weighing approximately 20.0 g, was extracted by maceration in 150 mL of ethanol at room temperature twice, yielding approximately 277.8 to 329.8 mg of extract. This *P. albidus* ethanol extract (PE) was used for biological activities.

### 4.3. Quantitative Analysis for Theophylline, Theobromine, EGCG, and Caffeine

#### 4.3.1. Reagents and Analytical Instruments

Theophylline, theobromine, epigallocatechin gallate (EGCG), and caffeine reference standards were obtained from Sigma-Aldrich (St. Louis, MO, USA). Methanol (MeOH), water, and formic acid (LC–MS grade) were sourced from Daejung (Ulsan, Republic of Korea) and Daejung Chemicals & Metals Co., Ltd. (Siheung, Republic of Korea), respectively. Four different P. albidus (māmaki) extracts were used for quantitative analysis, and green tea powder from O’Sulloc (Jeju, Republic of Korea) was extracted with 100% ethanol to prepare the control extract.

#### 4.3.2. LC-MS/MS Instruments and Conditions

Quantitative analysis was conducted using an Agilent 1200 Series HPLC system equipped with a diode array detector and a 6130 Series electrospray ionization (ESI) mass spectrometer (Agilent Technologies). Chromatographic separation was achieved on an analytical Kinetex C18 100 Å column (100 × 2.1 mm, 5 μm; Phenomenex) maintained at 30 °C, with a flow rate of 0.3 mL/min. The mobile phase consisted of solvent A (water containing 0.1% formic acid) and solvent B (methanol). Gradient conditions were optimized for each analyte: 7–8% B for theobromine, 11–13% B for theophylline, 17–18% B for caffeine, and 23–25% B for EGCG. Each analytical run lasted 20–21 min, followed by column washing with 100% methanol and re-equilibration with 10% methanol. The injection volume was 10 µL. Mass spectrometric detection was performed in Selected Ion Monitoring (SIM) mode, using positive ionization for theobromine (*m*/*z* 181.1), theophylline (*m*/*z* 181.1), and caffeine (*m*/*z* 195.1) and negative ionization for EGCG (*m*/*z* 457.0). The needle wash time was set to 15 s between injections.

#### 4.3.3. Preparation of the Calibration Curve and Sample Pretreatment

Stock solutions (1 mg/mL) of theobromine, theophylline, caffeine, and EGCG were prepared by dissolving each reference standard in LC–MS-grade methanol and water (60:40, *v*/*v*). Working standard solutions for calibration curve construction were prepared at concentrations ranging from 20 to 10,000 ppb, depending on the analyte. Each calibration level was analyzed in triplicate, and calibration curves were generated by plotting peak area (Y) against nominal concentration (X, ppb). Four different *P. albidus* (māmaki) extracts were reconstituted in LC–MS-grade methanol to final concentrations ranging from 0.5 to 1.0 mg/mL, depending on the target compound. A commercially available green tea extract was similarly prepared at concentrations of 0.05–0.5 mg/mL and used as a positive control. Prior to LC–MS analysis, all *P. albidus* and green tea extract solutions were vortexed, sonicated for 10 min, and filtered through 0.45 µm PTFE syringe filters. To enhance EGCG stability, 0.1% formic acid was added to the extraction solvent, adjusting the solution to an acidic pH known to improve EGCG preservation.

### 4.4. Antioxidant Assay

#### 4.4.1. 2,2-Diphenyl-1-Picrylhydrazyl (DPPH) Radical-Scavenging Assay

Total antioxidant capacity (TAC) is a key parameter in food quality assessment, as naturally occurring factors can lead to substantial variation in antioxidant levels. In this study, the Trolox Equivalent Antioxidant Capacity (TEAC) of *P. albidus* extract was determined by comparing its ability to reduce the stable free-radical DPPH to those of Trolox, a synthetic antioxidant standard. DPPH is a nitrogen-centered radical that exhibits a deep purple color, becoming colorless upon reduction. Trolox standard solutions were prepared using an assay kit by diluting Trolox with Reagent A from the kit at a 1:10 ratio (100 μL of Trolox and 900 μL of Reagent A). Serial mixtures of diluted standard solutions with Reagent A were used to generate the TEAC value, with combinations of 0 µL, 10 µL, and 20 µL of standard solution with 100 µL, 90 µL, and 80 µL of Reagent A corresponding to TEAC values of 0, 100, and 200, respectively. For sample analysis, 100 µL of each extract was combined with 150 μL of DPPH solution in a 96-well microplate. Absorbance was measured at 500 nm every 5 min over a 2 h period at room temperature using an HTS 7000 Bio Assay Reader (Perkin Elmer, Norwalk, CT, USA). All measurements were performed in triplicate. The percentage of radical-scavenging activity was calculated using the following formula:

$$Scavenging\;effect\;(\%)=\frac{\left[{Abs}_{control}-\left({Abs}_{sample}-{Abs}_{background}\right)\right]}{{Abs}_{control}}\times 100\%$$

#### 4.4.2. Ferric Reducing Ability of Plasma (FRAP) Assay

The FRAP assay quantified antioxidant activity based on the ability of tested compounds to reduce Fe^3+^ to Fe^2+^. In the presence of the working FRAP reagent, the formation of Fe^2+^ produces an intense blue complex, providing a measurable indicator of antioxidant capacity. To prepare the components, 0.054 g of FeCl_3_ was dissolved in 10 mL of distilled water to obtain a 20 mM ferric chloride solution. Separately, 0.031 g of solid TPTZ (2,4,6-tri [2-pyridyl]-s-triazine) was mixed with 10 mL of a pre-prepared 40 mM HCl solution to obtain a 10 mM TPTZ solution. The working FRAP solution was prepared by combining 3 mL of 20 mM ferric chloride, 3 mL of 10 mM TPTZ, and 30 mL of a pre-prepared 300 mM acetate buffer (pH 3.6). Standards were then prepared from a 4 mM FeSO_4_ solution generated by dissolving 11.12 mg of FeSO_4_ with 10 mL of distilled water. *P. albidus* samples were analyzed following the FRAP protocol. In a 96-well plate, each standard and *P. albidus* samples were tested in triplicate. For each well, 10 μL of the standards and samples were combined with 250 μL of the working FRAP solution. The plate was incubated at 37 °C for 4 min and mixed prior to measurement. Absorbance was then recorded at 593 nm for 1 h using a microplate reader.

#### 4.4.3. Oxygen Radical Absorbance Capacity (ORAC) Assay

The antioxidant activity (AOA) of the samples was evaluated using the ORAC (oxygen radical absorbance capacity) assay following the manufacturer’s protocol (Cell Biolabs, San Diego, CA, USA). Trolox standard solutions were prepared at concentrations of 20, 40, 80, 200, and 400 μM. For each measurement, 25 μL of Trolox standard, crude extract, or extraction solvent (blank) was added to the designated wells of a 96-well microplate, followed by the addition of 200 μL of 100 nM fluorescein (FL) solution. The plate was then sealed and incubated at 37 °C for 20 min in a Victor multilabel microplate reader (PerkinElmer, Turku, Finland). Free-radical generation was initiated by adding 35 μL of 0.36 M 2,2′-azobis(2-methylpropionamidine) dihydrochloride (AAPH) solution to each well. Fluorescence was measured at one-minute intervals using an excitation wavelength of 485 nm and an emission wavelength of 535 nm, continuing until the fluorescence intensity decreased to less than 5% of the initial value. All standards and samples were analyzed in duplicate.

### 4.5. Suppression of IL-8 Expression by qPCR Analysis

#### 4.5.1. Cell Culture and Treatment

Human Aortic Endothelial Cells (HAECs) were cultured in endothelial growth media supplemented with 10% Fetal Bovine Serum (FBS), 10 mM L-glutamine, 1% penicillin/streptomycin, and 1 μg/mL hydrocortisone. Cells were maintained in Cellstar^®^ Filtered Cap 75 cm^2^ cell-culture treated screw cap flasks and incubated at 37 °C in a humidified atmosphere containing 5% CO_2_. Subculturing was performed at 85–95% confluence and involved centrifugation, removal of the supernatant, and resuspension of the cell pellet in fresh medium.

#### 4.5.2. RNA Isolation

Total RNA was extracted using the ZYMO^®^ Research Quick-RNA™ MicroPrep kit (Zymo Research Corporation, Tustin, CA, USA) following the manufacturer’s protocol. After treatment and incubation, cells were lysed with RNA Lysis Buffer and pipette-mixed with 95–100% ethanol to facilitate RNA precipitation. The lysate–ethanol mixture was transferred to a ZYMO-Spin™ IC Column and centrifuged at 16,000 *g* for 30 s at 22 °C. RNA bound to the column matrix was purified using ZYMO^®^ Wash Buffer, DNA Digestion Buffer, and RNA Prep Buffer. Purified RNA was eluted in 15 μL of DNase/RNase-Free Water, and RNA concentration and purity were assessed using a Thermo Scientific™ Nanodrop (Thermo Fisher, Waltham, MA, USA).

#### 4.5.3. cDNA Synthesis

Following RNA isolation, cDNA synthesis reactions were prepared by combining 5 μL of 5× buffer, 1.25 μL of dNTP mix, 1.25 μL random primer, 0.625 μL of MMLV reverse transcriptase, and sample-specific volumes of RNase-Free Water and undiluted RNA. Each reaction mixture (25 μL total volume) was transferred to a 0.2 mL MicroAmp^®^ reaction tube with a cap and placed in an Applied Biosystems™ Veriti™ 96-Well Thermal Cycler for cDNA synthesis.

#### 4.5.4. Quantitative Real-Time Polymerase Chain Reaction (RT-PCR)

Following cDNA synthesis, gene expression was assessed for IL-8, using GAPDH as the housekeeping gene. A qPCR master mix was prepared by combining 10 μL of Power SYBR^®^ Green, 2 μL of 5 μM forward primer, 2 μL of 5 μM reverse primer, 5 μL of RNase Free Water, and 1 μL of cDNA per reaction. The mixture was dispensed in duplicates into a MicroAmp^®^ Fast 96-well reaction plate (0.1 mL). Amplification was performed on an Applied Biosystems™ QuantStudio3™ real-time PCR system for 40 cycles, consisting of a 95 °C phase for 15 s, a 58 °C phase for 1 min, and a 60 °C phase for 15 s. Gene expression levels were quantified using comparative threshold (Ct) analysis and normalized to the housekeeping GAPDH gene (Table S3).

### 4.6. Statistical Analysis

One-way ANOVA was performed using GraphPad Prism 5 to assess statistically significant differences in group means. Post hoc comparisons were conducted using Tukey’s HSD test. Statistical significance was defined as *p* < 0.05.

## 5. Conclusions

The phytochemical investigation of *P. albidus* leaves confirms the presence of a diverse array of bioactive phenolic constituents, including cinchonain-type flavanolignans, flavone *C*-glycosides, phenylpropanoids, and cinnamic and benzoic acid derivatives. Comparative LC-MS/MS analysis with green tea further establishes *P. albidus* as chemically distinct, confirming that it is a stimulant-free botanical lacking detectable levels of caffeine, theophylline, theobromine, and EGCG. While isolated constituents such as vitexin (**6**), cinchonain Ia (**2**), and (7*S*,8*R*)-dihydrodehydro diconiferyl alcohol (**11**) displayed strong free radical-scavenging and reducing abilities in cell-free antioxidant assays, evaluation of IL-8 mRNA expression in TNF-α-stimulated HAECs revealed no statistically significant reduction in IL-8 mRNA expression by the crude plant extract (PE) or vitexin (**6**) compared to the control (*p* < 0.05). Importantly, the crude extract, representing the traditional tea infusion matrix, exhibited neither measurable therapeutic anti-inflammatory suppression nor adverse pro-inflammatory elevation of IL-8 levels under the tested conditions. Conversely, isolated compounds **3** and **9** significantly increased IL-8 mRNA levels (*p* < 0.05), identifying potential pro-inflammatory and toxicological risks associated with concentrated individual constituents. Overall, the primary value of *P. albidus* lies in its distinct phytochemical composition and chemical antioxidant properties, while potential health or safety must carefully consider the cellular anti-inflammatory efficacy and the potential risks of specific pro-inflammatory constituents. Further in vitro and in vivo studies and pharmacokinetic evaluations are warranted to translate these findings toward clinical relevance and to strengthen the bridge between ethnobotanical knowledge and therapeutic development.

## Figures and Tables

**Figure 1:**
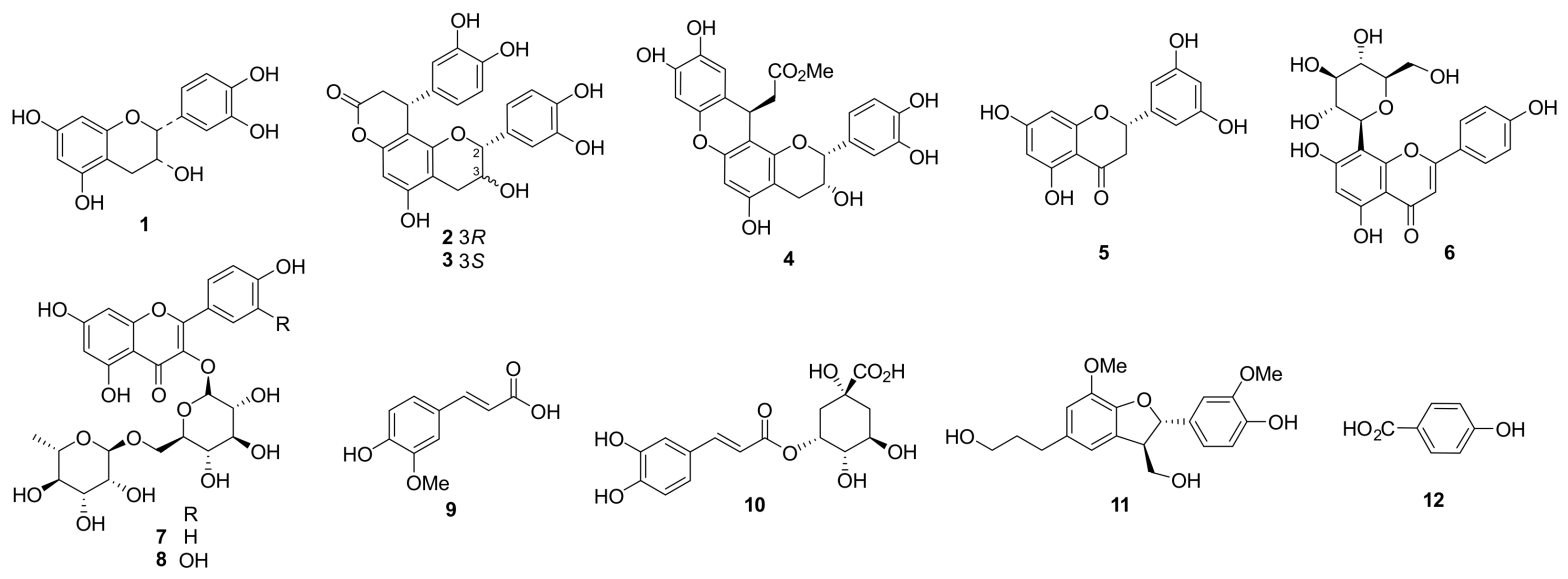
Compounds isolated from the leaves of *P. albidus*.

**Figure 2:**
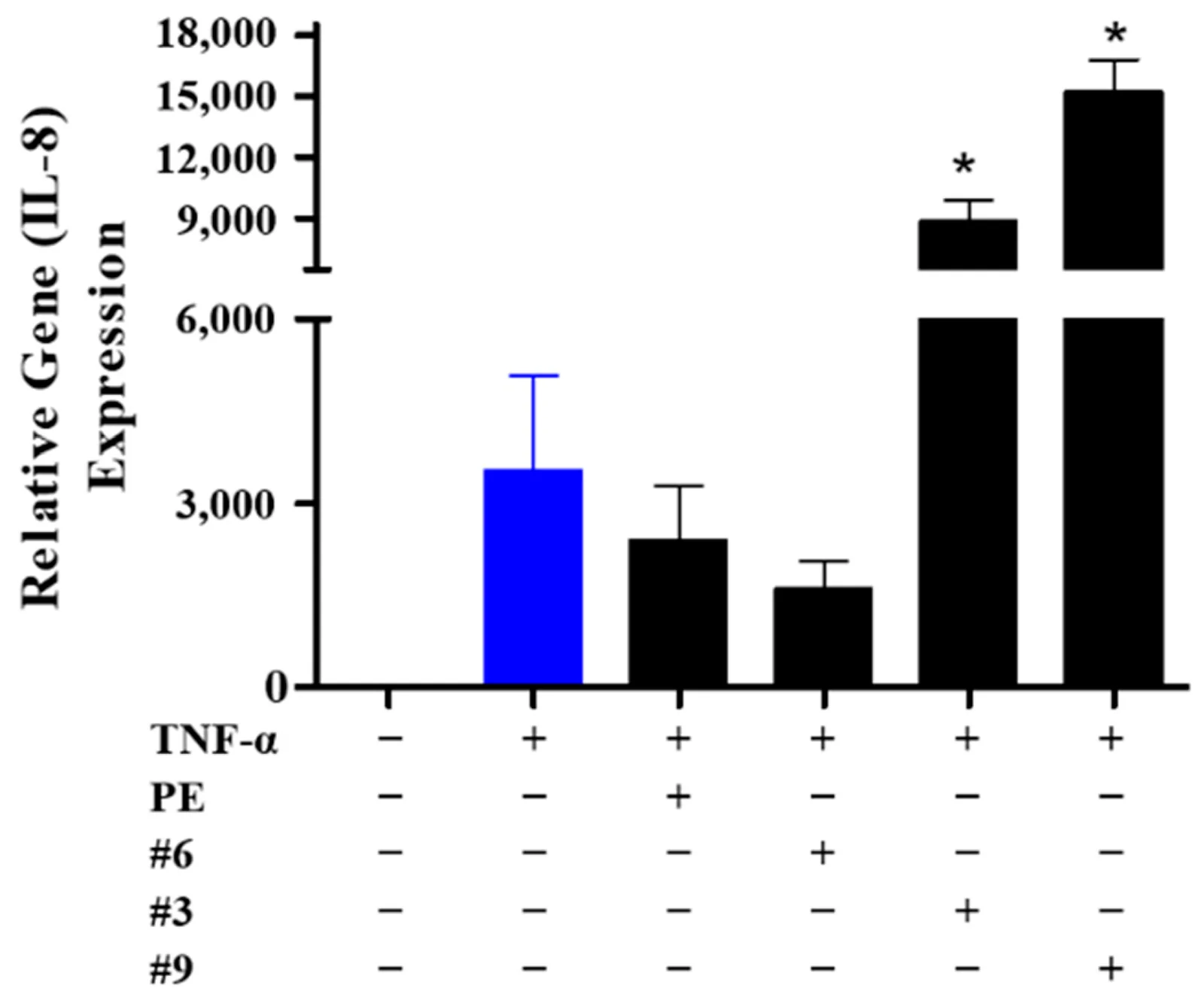
Effects of *P. albidus* ethanol extract (PE) and isolated compounds on TNF-α-induced expression of the IL-8 pro-inflammatory gene. HAECs were incubated with *P. albidus* ethanol extract (PE) and isolated compounds for 30 min prior to the addition of co-treatment with TNF-α (10 ng/mL) for 24 h. Gene expression was normalized using beta-actin as the housekeeping gene. The expression of IL-8 genes was measured. Data are represented as mean ± SEM (*n* = 3–4, * *p* < 0.05 compared to the TNF-α-stimulated control group).

**Figure 3:**
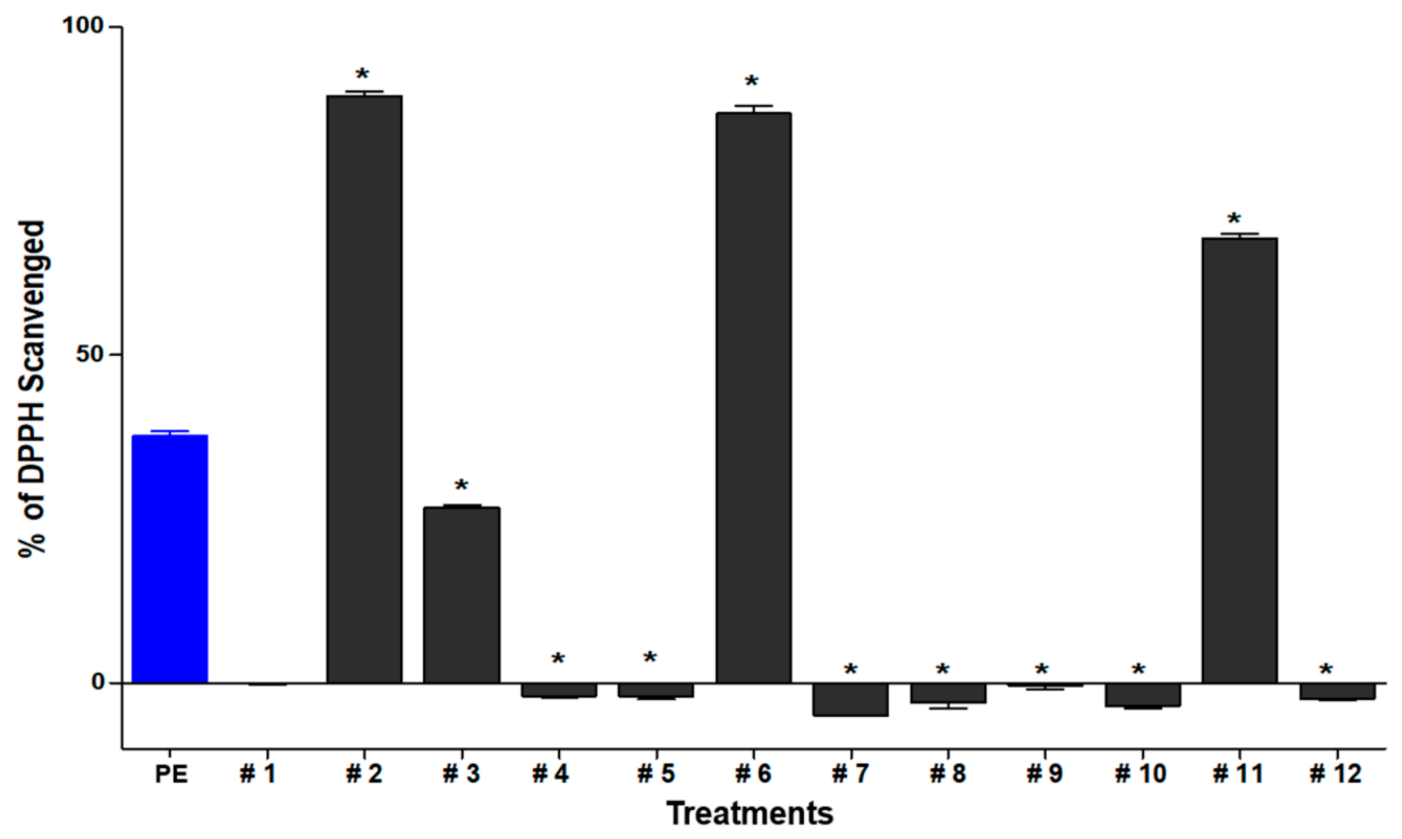
DPPH free radical-scavenging activity of isolated compounds from *P. albidus*. The DPPH radical-scavenging activity of each compound (10 µg/mL) was determined, and all samples were analyzed in triplicate. The scavenging rate was calculated. A higher scavenging rate indicates stronger DPPH free radical-scavenging activity. Data are represented as mean + SEM. (* *p* < 0.05 vs. crude extract (PE) group).

**Figure 4:**
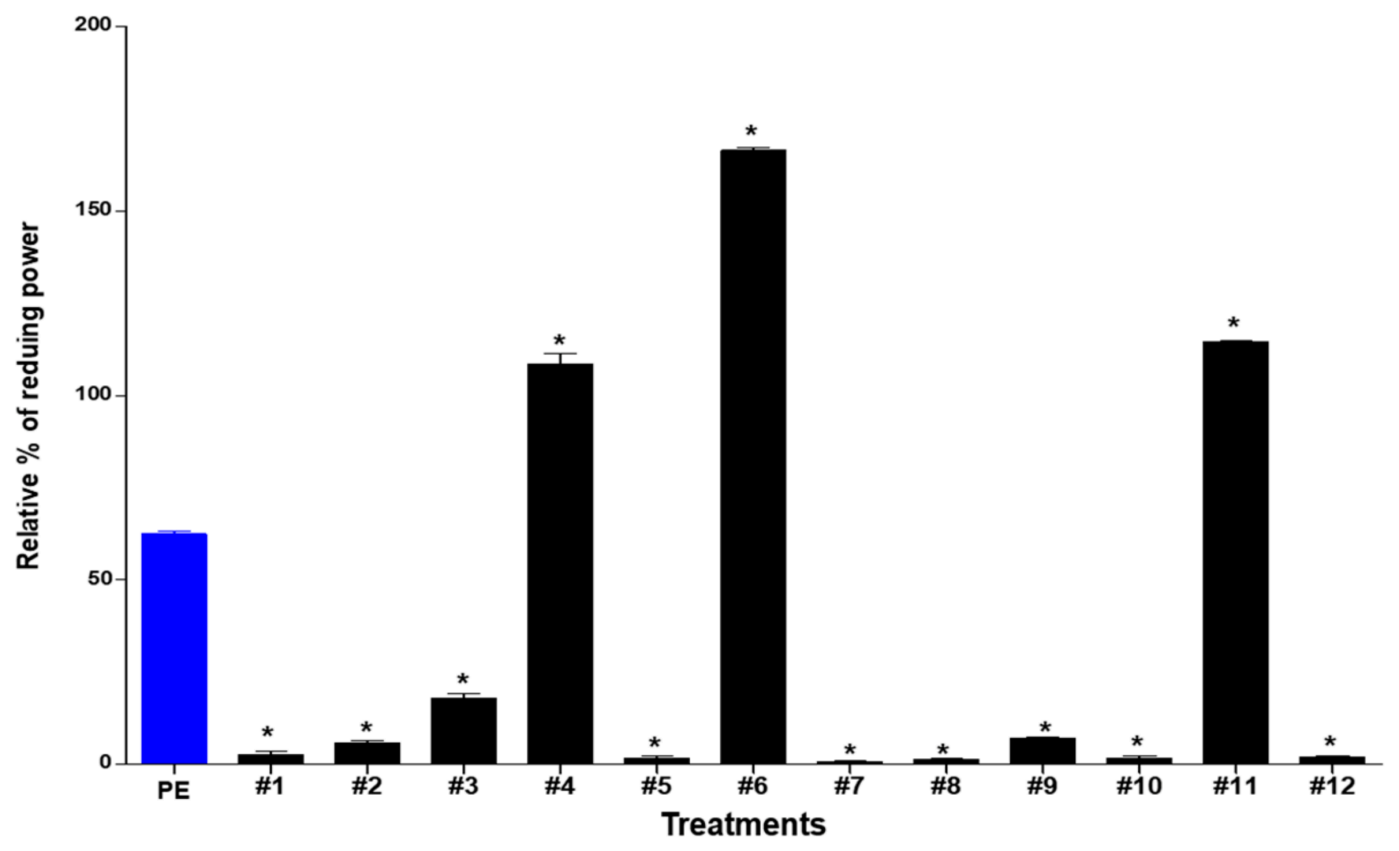
Ferric reducing power of isolated compounds from *P. albidus*. The ferric reducing antioxidant power (FRAP) of each compound (10 µg/mL) was evaluated based on their ability to reduce Fe^3+^ to Fe^2+^. Absorbance was measured at the appropriate wavelength after incubation with the FRAP reagent; higher absorbance indicates greater reducing power. Data are represented as mean + SEM. (*n* = 3, * *p* < 0.05 vs. crude extract (PE) group).

**Figure 5:**
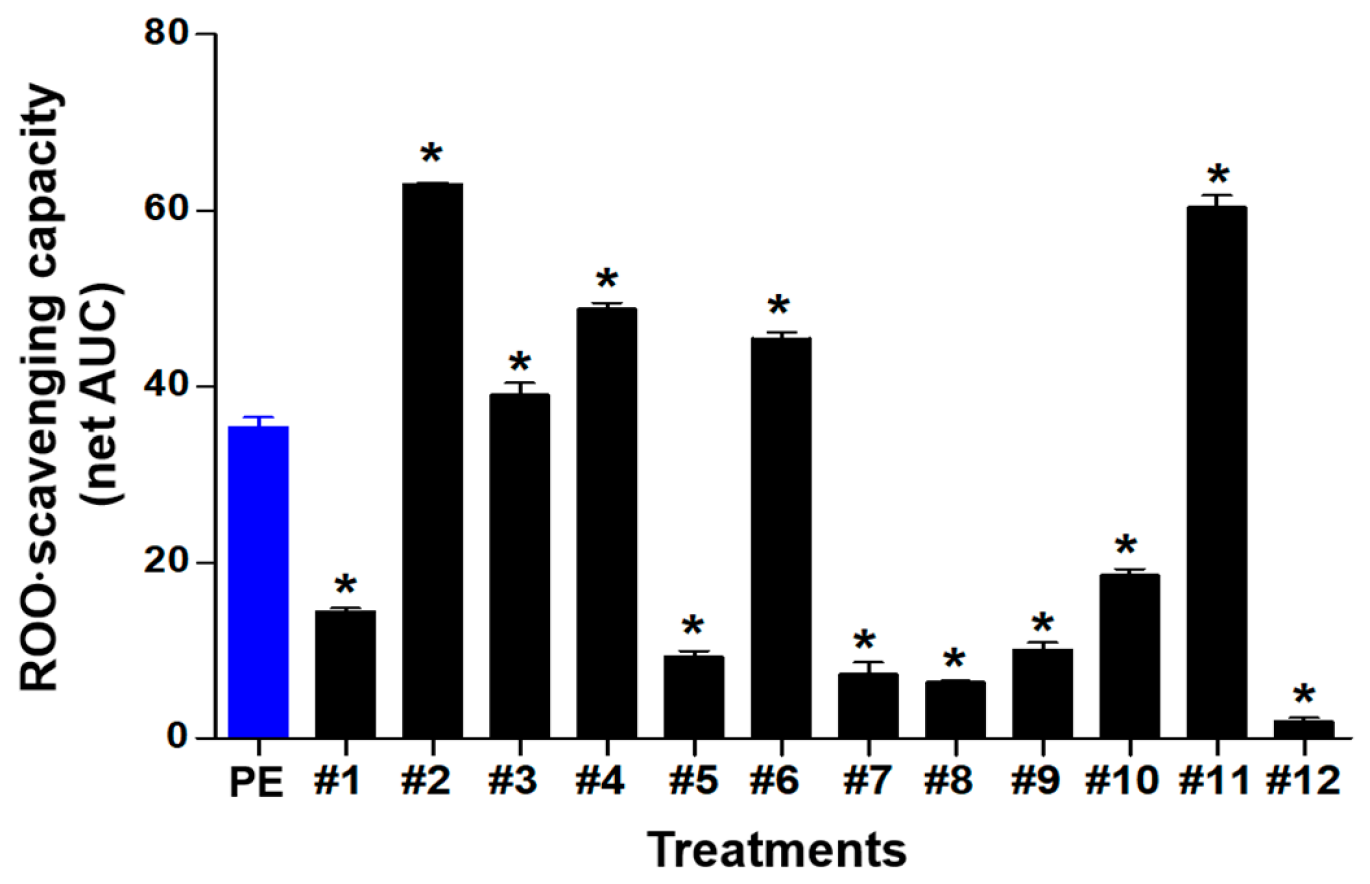
ROO^•^ free radical-scavenging activity of compounds isolated from *P. albidus*. AAPH was used to produce ROO radicals, which can bleach fluorescent intensity. AUC values of each compound (5 µg/mL) in the ORAC assay were determined. Data are represented as mean + SEM. (*n* = 3, * *p* < 0.05 vs. crude extract (PE) group).

## Data Availability

Data will be made available upon request.

## Supplementary Materials

The following supporting information can be downloaded at: https://www.mdpi.com/article/10.3390/molecules31193449/s1, Figure S1: The ^1^H NMR spectrum of compound **1** (CD_3_OD, 850 MHz); Figure S2: The ESIMS data of **1** (positive-ion mode); Figure S3: The ^1^H NMR spectrum of compound **2** (CD_3_OD, 850 MHz); Figure S4: The ESIMS data of **2** (negative-ion mode); Figure S5: The ^1^H NMR spectrum of compound **3** (CD_3_OD, 850 MHz); Figure S6: The ESIMS data of **3** (negative-ion mode); Figure S7: The ^1^H NMR spectrum of compound **4** (CD_3_OD, 850 MHz); Figure S8: The ESIMS data of **4** (positive-ion mode); Figure S9: The ^1^H NMR spectrum of compound **5** (CD_3_OD, 850 MHz); Figure S10: The ESIMS data of **5** (positive-ion mode); Figure S11: The ^1^H NMR spectrum of compound **6** (CD_3_OD, 850 MHz); Figure S12: The ESIMS data of **6** (positive-ion mode); Figure S13: The ^1^H NMR spectrum of compound **7** (CD_3_OD, 850 MHz); Figure S14: The ESIMS data of **7** (negative-ion mode); Figure S15: The ^1^H NMR spectrum of compound **8** (CD_3_OD, 850 MHz); Figure S16: The ESIMS data of **8** (negative-ion mode); Figure S17: The ^1^H NMR spectrum of compound **9** (CD_3_OD, 850 MHz); Figure S18: The ESIMS data of **9** (negative-ion mode); Figure S19: The ^1^H NMR spectrum of compound **10** (CD_3_OD, 850 MHz); Figure S20: The ESIMS data of **10** (negative-ion mode); Figure S21: The ^1^H NMR spectrum of compound **11** (CD_3_OD, 850 MHz); Figure S22: The ESIMS data of **11** (negative-ion mode); Figure S23: The ^1^H NMR spectrum of compound **12** (CD_3_OD, 850 MHz); Figure S24: The ESIMS data of **12** (negative-ion mode); Table S1: Calibration curves of theophylline, theobromine, EGCG, and caffeine; Table S2: Concentrations of theophylline, theobromine, EGCG, and caffeine in *P. albidus* extracts and green tea extracts; Table S3: Primers and Sequences.

## Abbreviations

The following abbreviations are used in this manuscript:

DPPH	2,2-diphenyl-1-picrylhydrazyl
FRAP	Ferric reducing antioxidant power
ORAC	Oxygen radical absorbance capacity
HAECs	Human aortic endothelial cells
EGCG	Epigallocatechin gallate
NF-κB	Nuclear factor-kappa
TH	Tyrosine hydroxylase
TPH	Tryptophan hydroxylase
ROS	Reactive oxygen species
IL-8	Interleukin-8
TNF-α	Tumor necrosis factor-alpha
MAPKs	Mitogen-activated protein kinases
HAT	Hydrogen atom transfer
SET	Single-electron transfer
SAR	Structure-activity relationship
SIM	Selected Ion Monitoring
TPTZ	2,4,6-tripyridyl-s-triazine
AAPH	2,2′-azobis(2-amidinopropane) dihydrochloride
DHCA	Dihydrocaffeic acid
LC–MS/MS	Liquid Chromatography with Tandem Mass Spectrometry
HPLC	High-Performance Liquid Chromatography
UV	Ultraviolet
NMR	Nuclear magnetic resonance
TLC	Thin-layer chromatography
CC	Column chromatography
cDNA	Complementary deoxyribonucleic acid
qPCR	Quantitative polymerase chain reaction
RT-PCR	Realtime polymerase chain reaction
Ct	Comparative threshold
