# Supplementary material for: Evaluation of Interleukin-8 (IL-8) Expression in Human Aortic Endothelial Cells (HAECs) and Antioxidant Activity of Isolated Compounds from the Endemic Hawaiian Plant Pipturus albidus (Māmaki)
Source: Molecules. 2026 Sep 28;31(19):3449. doi: 10.3390/molecules31193449 (PMC13666199; doi:10.3390/molecules31193449)
Supplement: Supplementary file 1 [file molecules-31-03449-s001.zip › molecules-4543878-supplementary.pdf]

Evaluation of Interleukin-8 (IL-8) Expression in Human Aortic Endothelial Cells (HAECs) and Antioxidant Activity of Isolated Compounds from the Endemic Hawaiian Plant Pipturus albidus (Māmaki)

Pornphimon Meesakul [1,Δ], Kyung Ah Kim [2,Δ], Yaru Si [3,Δ], Yutaka Kuroki [4], Aya Wada [4], Kaitlin Villafuerte [1], Zhenquan Jia [3,*], Ki Hyun Kim [2,*] and Shugeng Cao [1,*]

[1] Department of Pharmaceutical Sciences, Daniel K. Inouye College of Pharmacy, University of Hawai'i at Hilo, 200 W. Kawili St., Hilo, HI 96720, USA; pmeesak@hawaii.edu (P.M.); kaitlin.villafuerte@gmail.com (K.V.)

[2] School of Pharmacy, Sungkyunkwan University, 2066 Seobu-Ro, Jangan-Gu, Suwon 16419, Republic of Korea.; ruddk5480@g.skku.edu

[3] Department of Biology, University of North Carolina at Greensboro, 321 Mciver St. 312 Greensboro, NC 27412, USA.; y_si@uncg.edu

[4] Delightex Pte. Ltd., 230 Victoria Street, #15-01/08 Bugis Junction Towers, Singapore 188024, Singapore; yutaka@delightexplorers.com (Y.K.); aya@delightexplorers.com (A.W.)

* Correspondence: z_jia@uncg.edu (Z.J.); khkim83@skku.edu (K.H.K.); scao@hawaii.edu (S.C.)

Δ These authors contributed equally.

Electronic Supplementary Information Contents:

**Figure S1**. The ^{1}H NMR spectrum of compound **1** (CD_3OD, 850 MHz)	3
**Figure S2**. The ESIMS data of **1** (positive-ion mode)	3
**Figure S3**. The ^{1}H NMR spectrum of compound **2** (CD_3OD, 850 MHz)	4
**Figure S4**. The ESIMS data of **2** (negative-ion mode)	4
**Figure S5**. The ^{1}H NMR spectrum of compound **3** (CD_3OD, 850 MHz)	5
**Figure S6**. The ESIMS data of **3** (negative-ion mode)	5
**Figure S7**. The ^{1}H NMR spectrum of compound **4** (CD_3OD, 850 MHz)	6
**Figure S8**. The ESIMS data of **4** (positive-ion mode)	6
**Figure S9**. The ^{1}H NMR spectrum of compound **5** (CD_3OD, 850 MHz)	7
**Figure S10**. The ESIMS data of **5** (positive-ion mode)	7
**Figure S11**. The ^{1}H NMR spectrum of compound **6** (CD_3OD, 850 MHz)	8
**Figure S12**. The ESIMS data of **6** (positive-ion mode)	8
**Figure S13**. The ^{1}H NMR spectrum of compound **7** (CD_3OD, 850 MHz)	9
**Figure S14**. The ESIMS data of **7** (negative-ion mode)	9
**Figure S15**. The ^{1}H NMR spectrum of compound **8** (CD_3OD, 850 MHz)	10
**Figure S16**. The ESIMS data of **8** (negative-ion mode)	10
**Figure S17**. The ^{1}H NMR spectrum of compound **9** (CD_3OD, 850 MHz)	11
**Figure S18**. The ESIMS data of **9** (negative-ion mode)	11
**Figure S19**. The ^{1}H NMR spectrum of compound **10** (CD_3OD, 850 MHz)	12
**Figure S20**. The ESIMS data of **10** (negative-ion mode)	12
**Figure S21**. The ^{1}H NMR spectrum of compound **11** (CD_3OD, 850 MHz)	13
**Figure S22**. The ESIMS data of **11** (negative-ion mode)	13
**Figure S23**. The ^{1}H NMR spectrum of compound **12** (CD_3OD, 850 MHz)	14
**Figure S24**. The ESIMS data of **12** (negative-ion mode)	14
**Table S1.** Calibration curves of theophylline, theobromine, EGCG, and caffeine	15
**Table S2.** Concentrations of theophylline, theobromine, EGCG, and caffeine in *P. albidus* extracts and green tea extracts	15
**Table S3.** Primers and Sequences	15

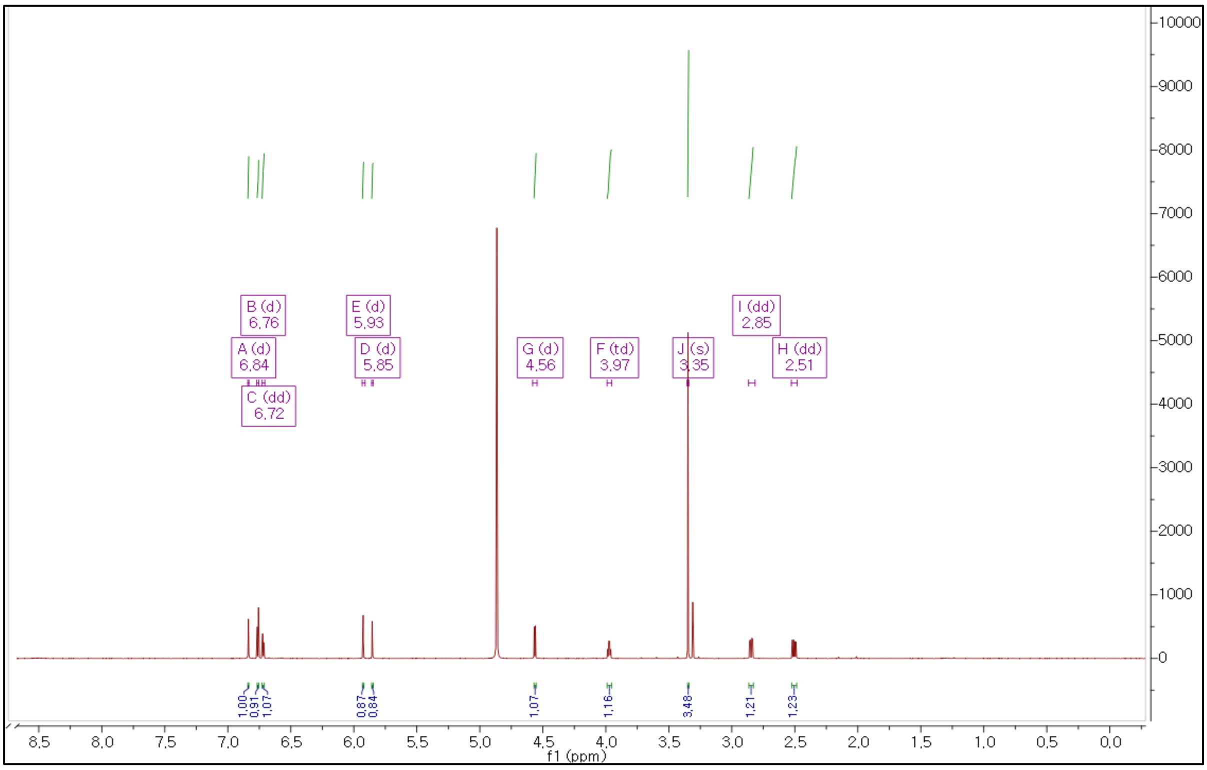

Figure S1. The ^{1}H NMR spectrum of compound **1** (CD_3OD, 850 MHz)

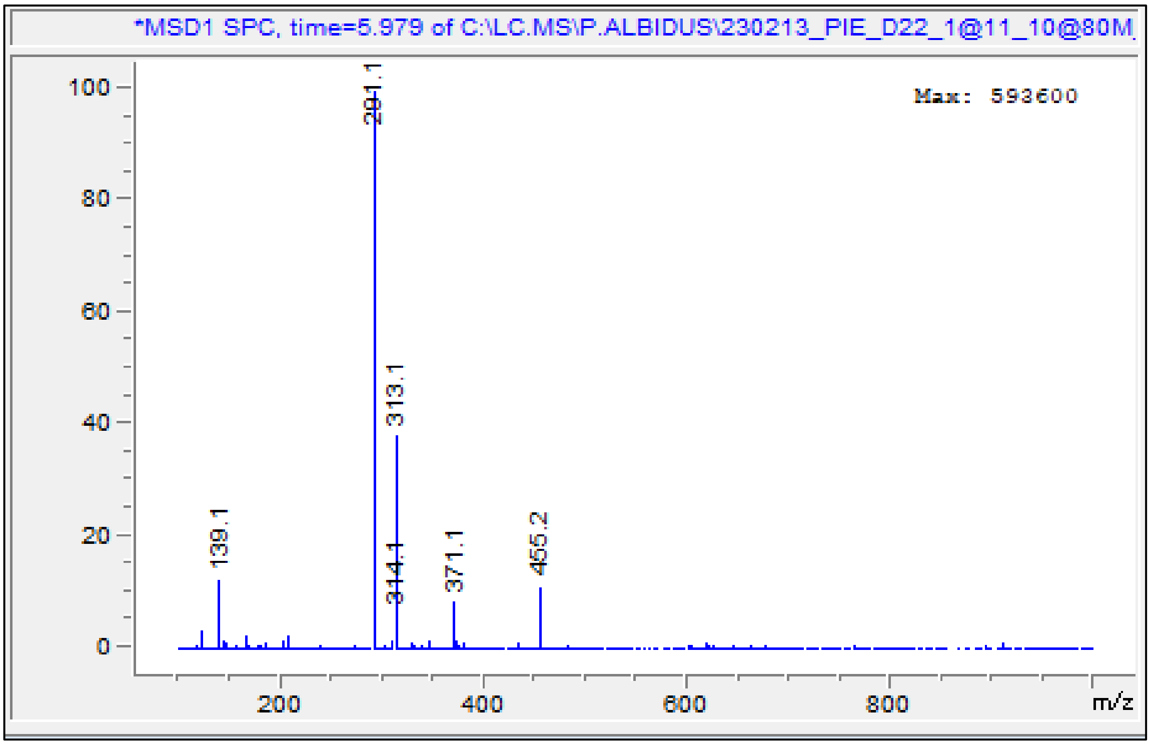

Figure S2. The ESIMS data of **1** (positive-ion mode)

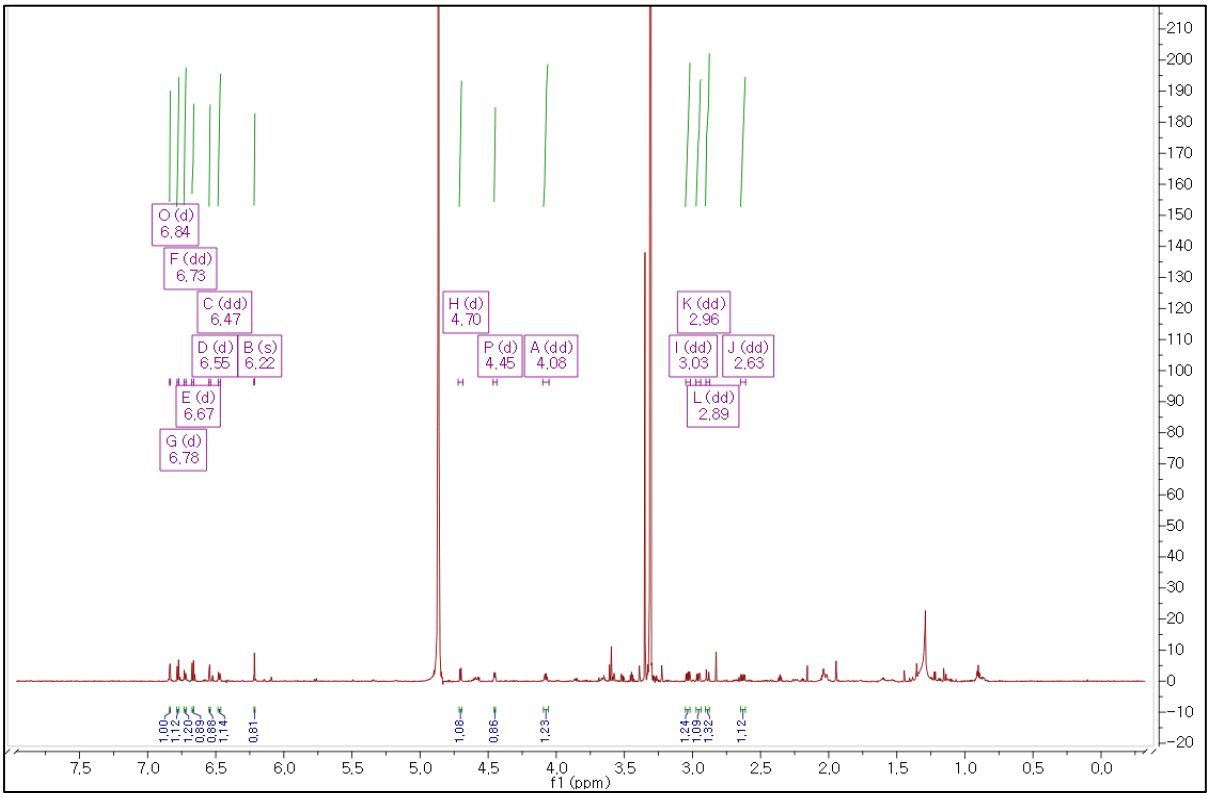

Figure S3. The ^{1}H NMR spectrum of compound **2** (CD_3OD, 850 MHz)

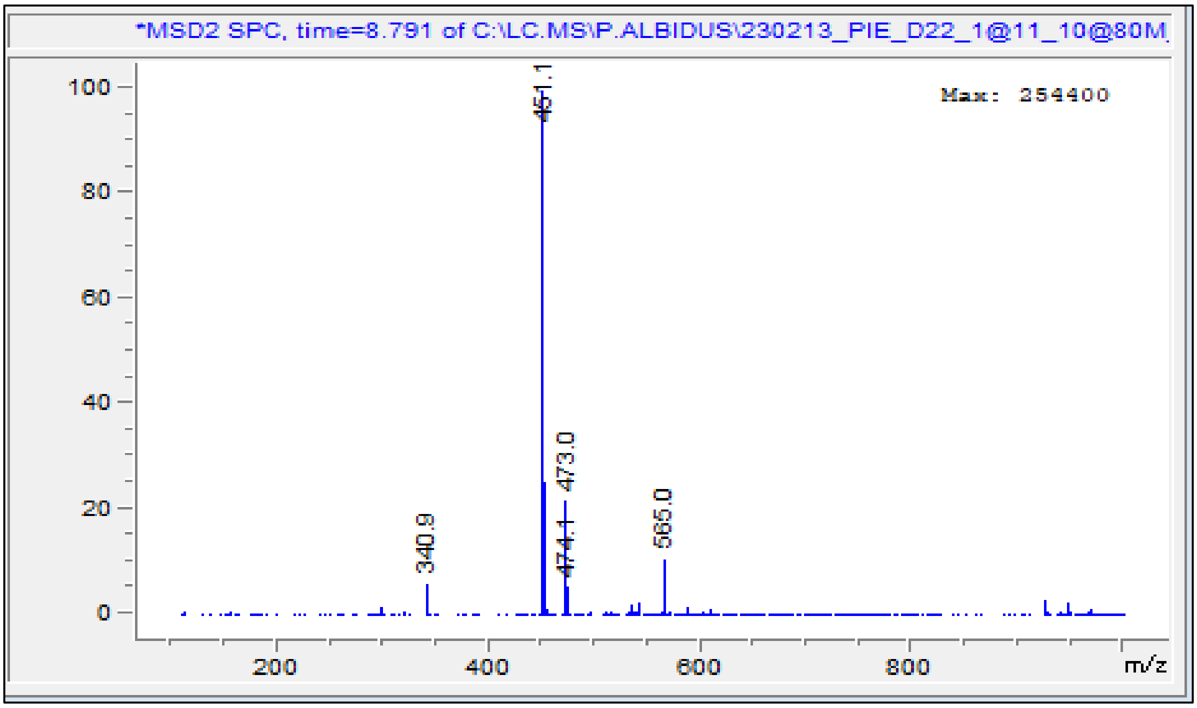

Figure S4. The ESIMS data of **2** (negative-ion mode)

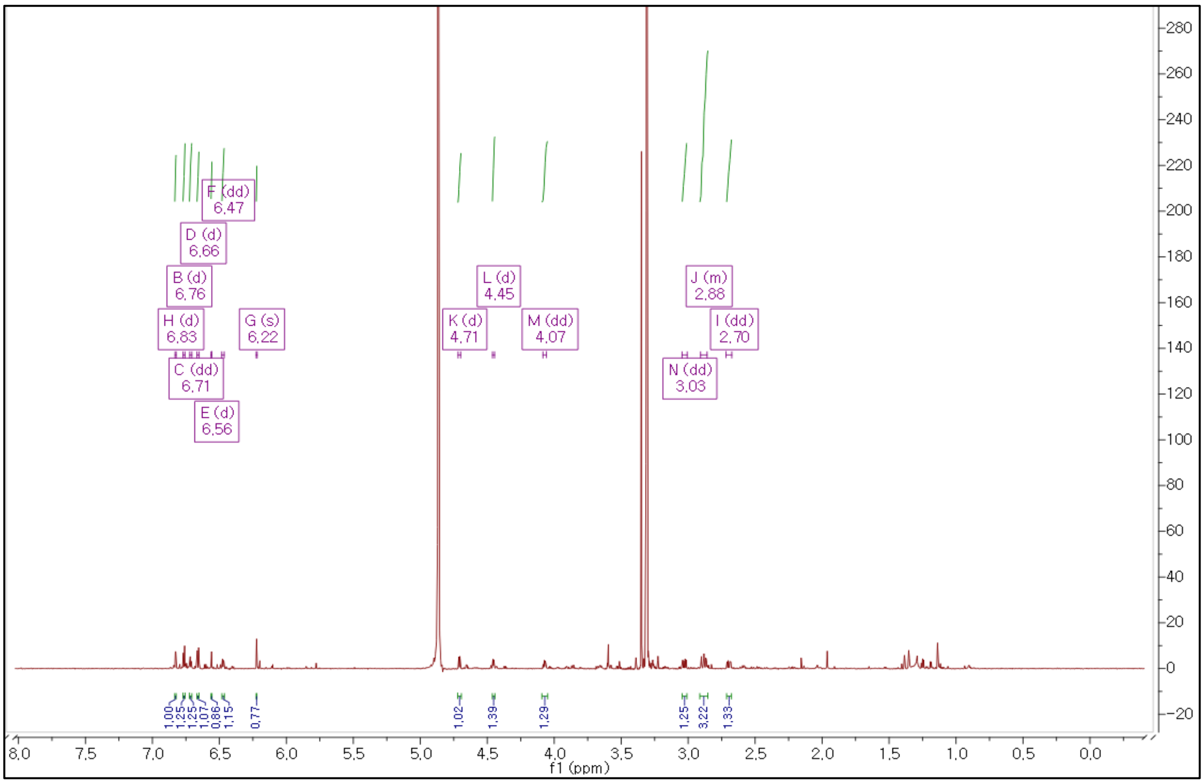

Figure S5. The ^{1}H NMR spectrum of compound **3** (CD_3OD, 850 MHz)

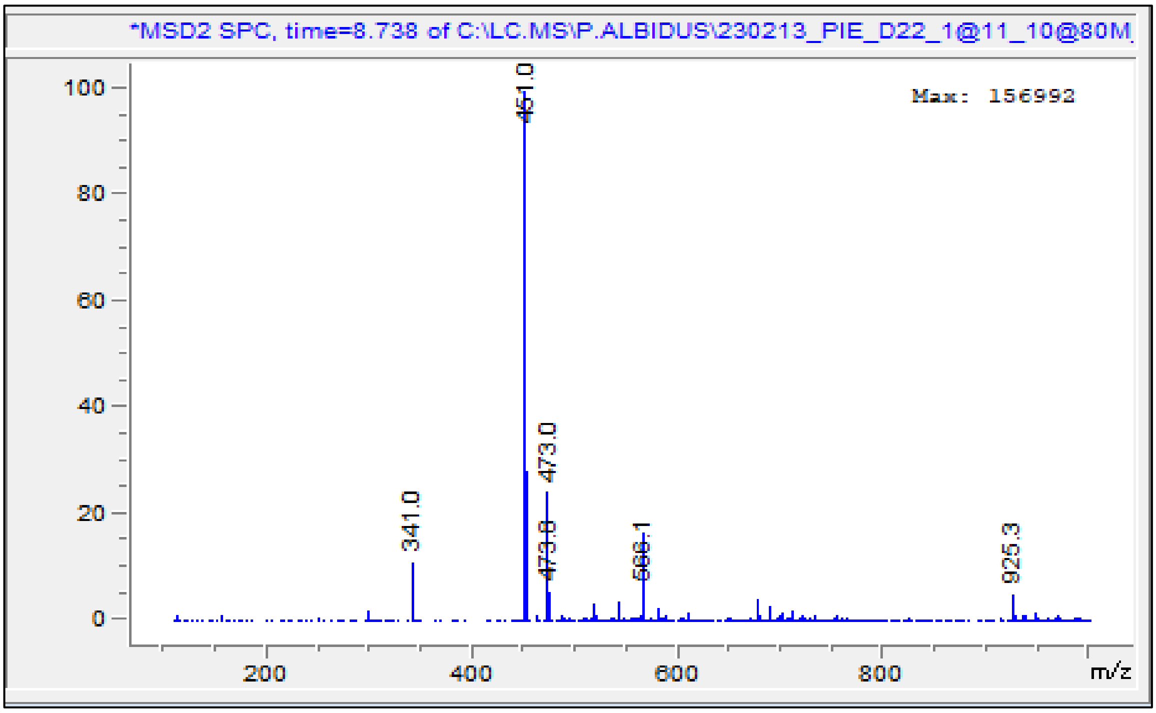

Figure S6. The ESIMS data of **3** (negative-ion mode)

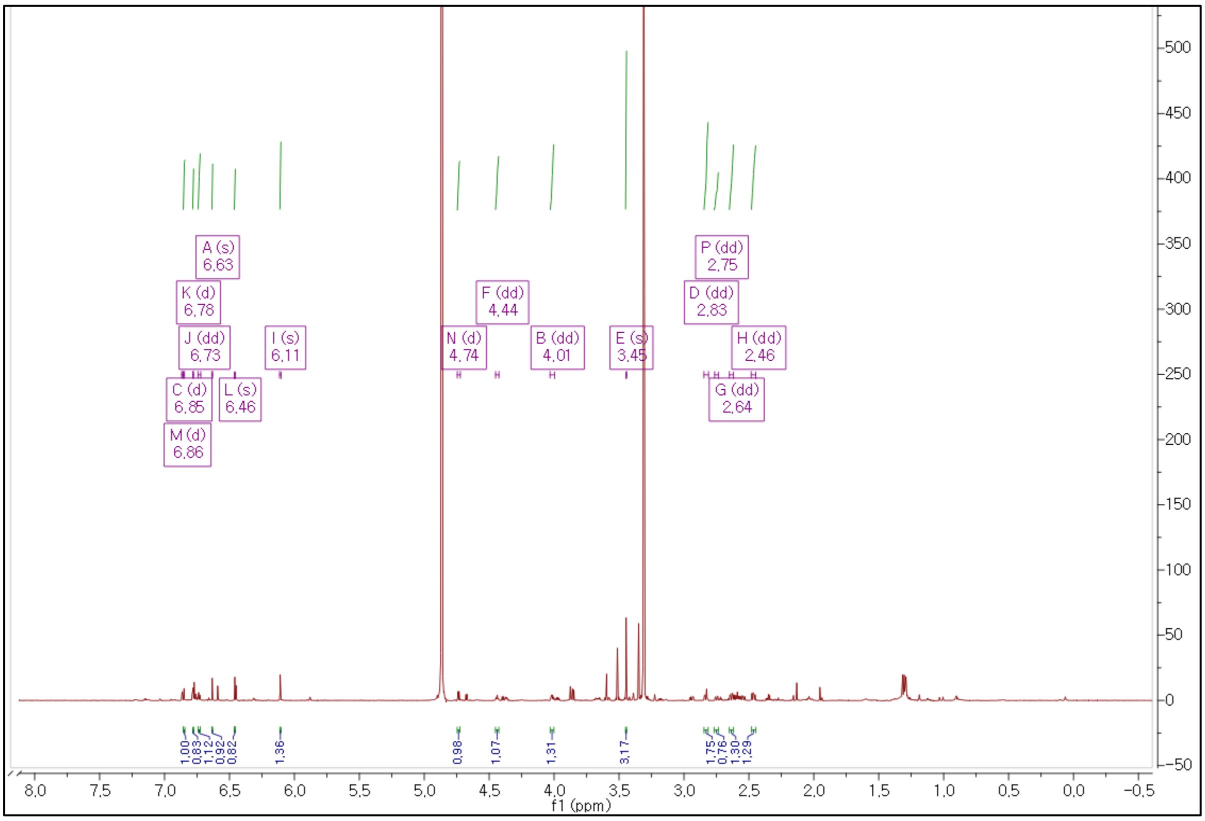

Figure S7. The ^{1}H NMR spectrum of compound **4** (CD_3OD, 850 MHz)

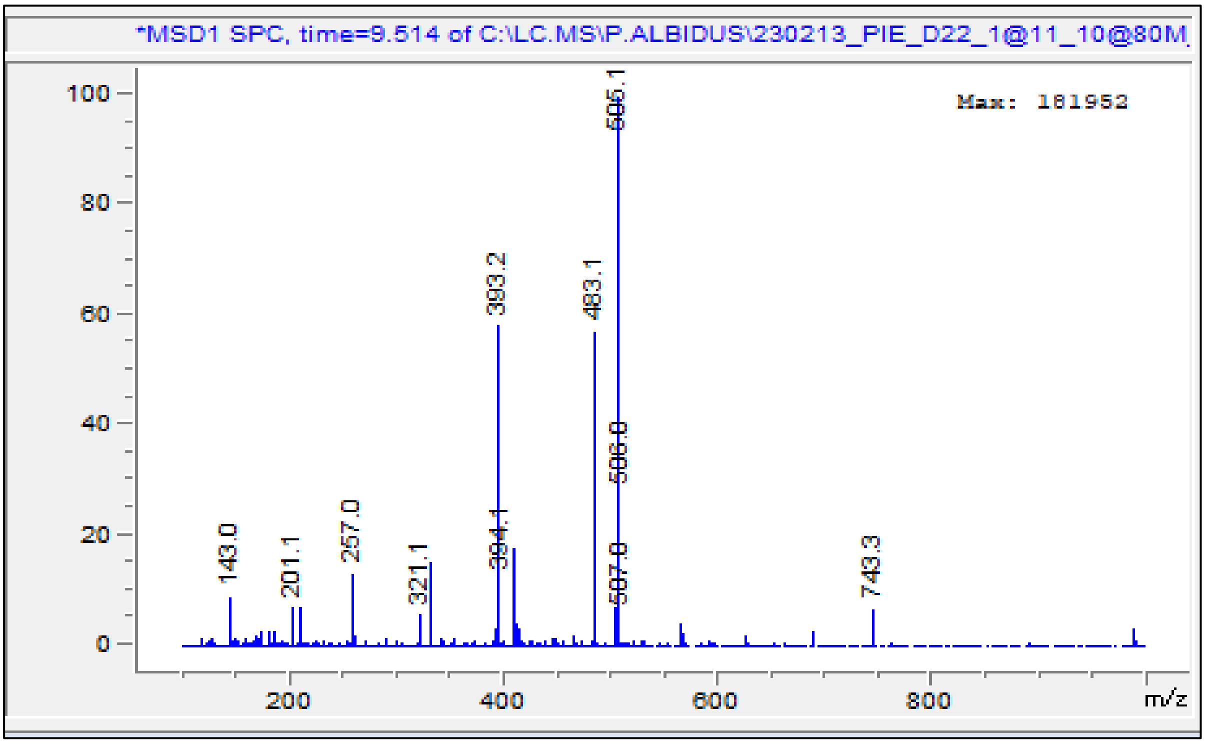

Figure S8. The ESIMS data of **4** (positive-ion mode)

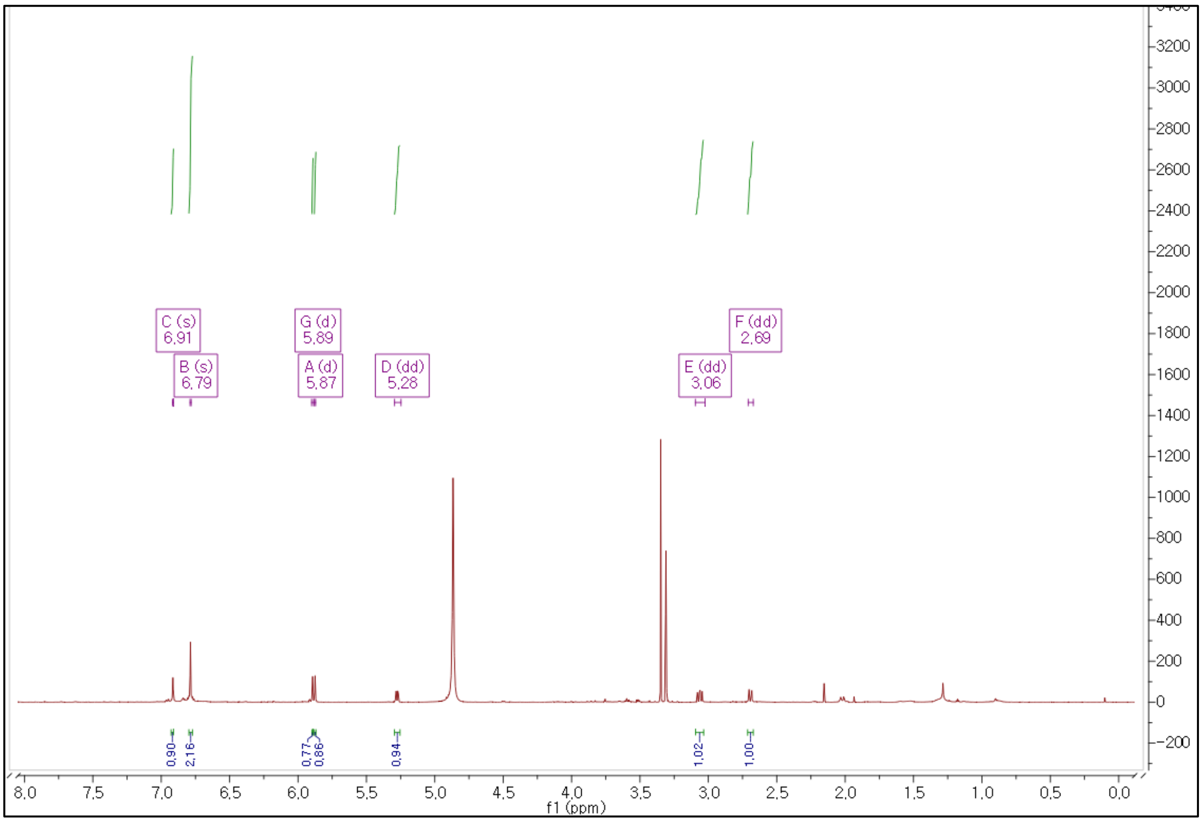

Figure S9. The ^{1}H NMR spectrum of compound **5** (CD_3OD, 850 MHz)

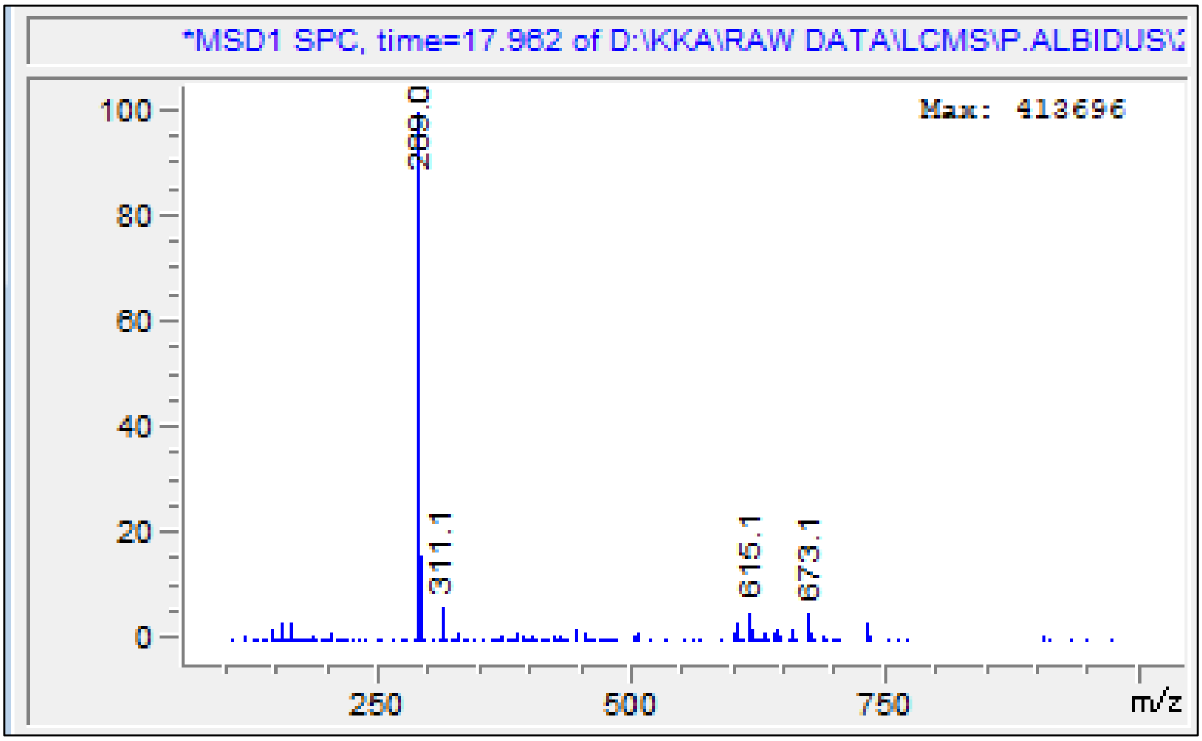

Figure S10. The ESIMS data of **5** (positive-ion mode)

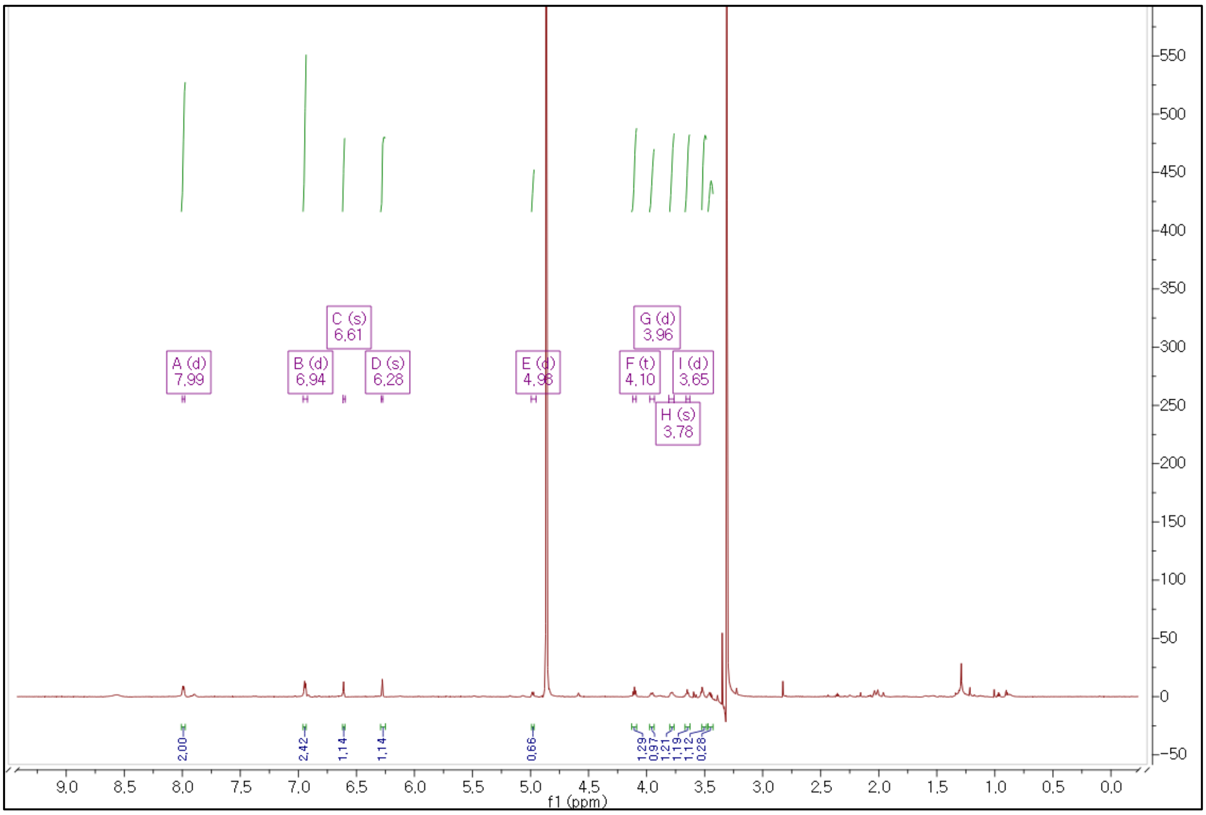

Figure S11. The ^{1}H NMR spectrum of compound **6** (CD_3OD, 850 MHz)

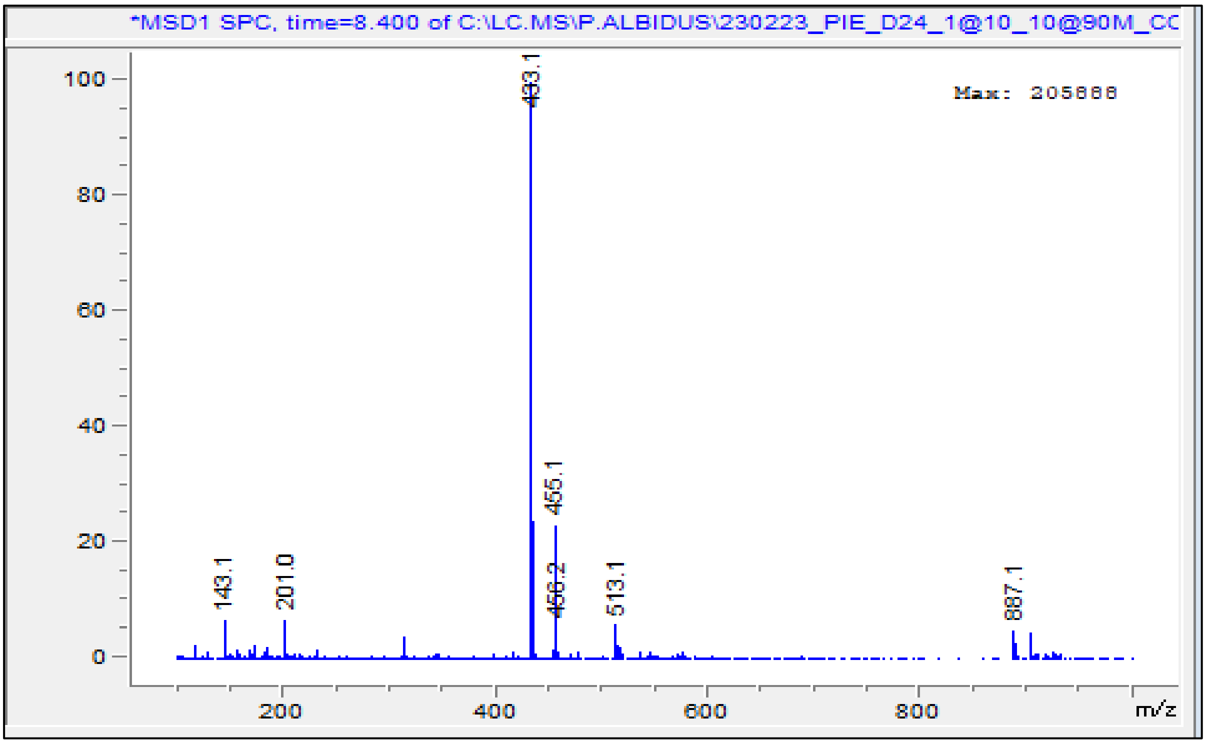

Figure S12. The ESIMS data of **6** (positive-ion mode)

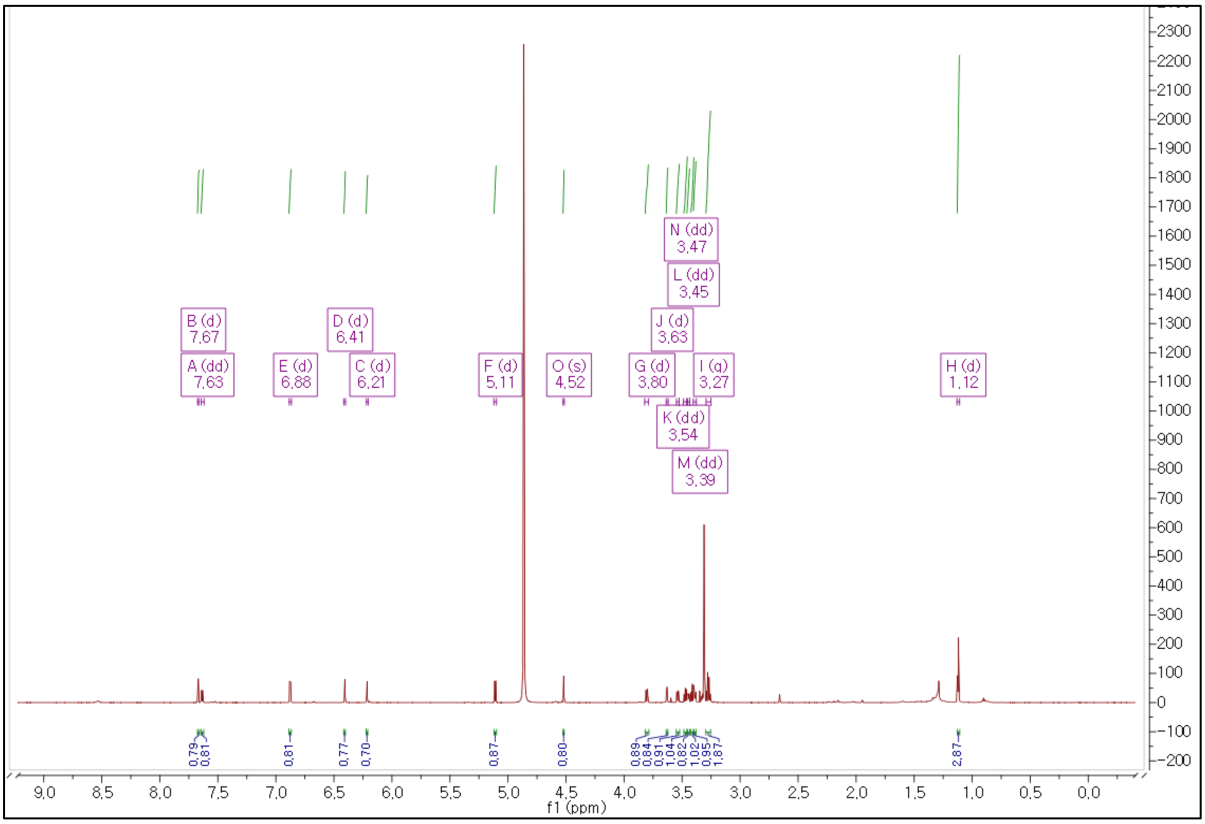

Figure S13. The ^{1}H NMR spectrum of compound **7** (CD_3OD, 850 MHz)

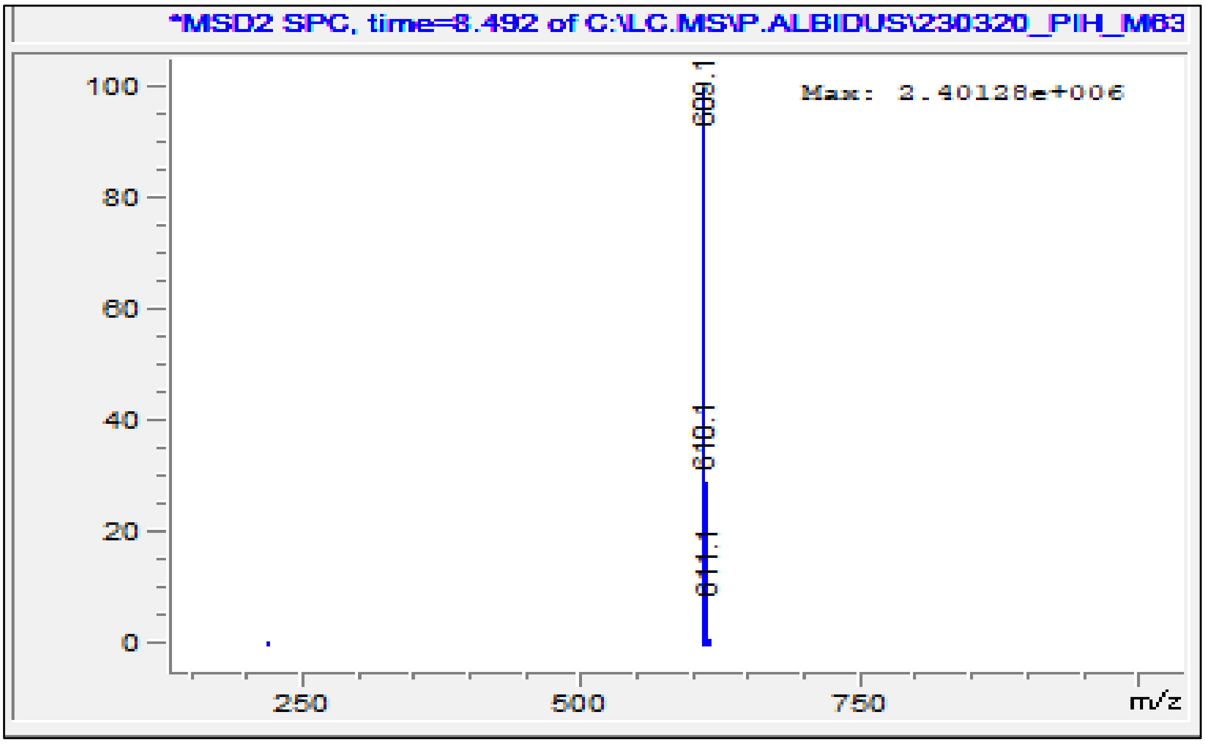

Figure S14. The ESIMS data of **7** (negative-ion mode)

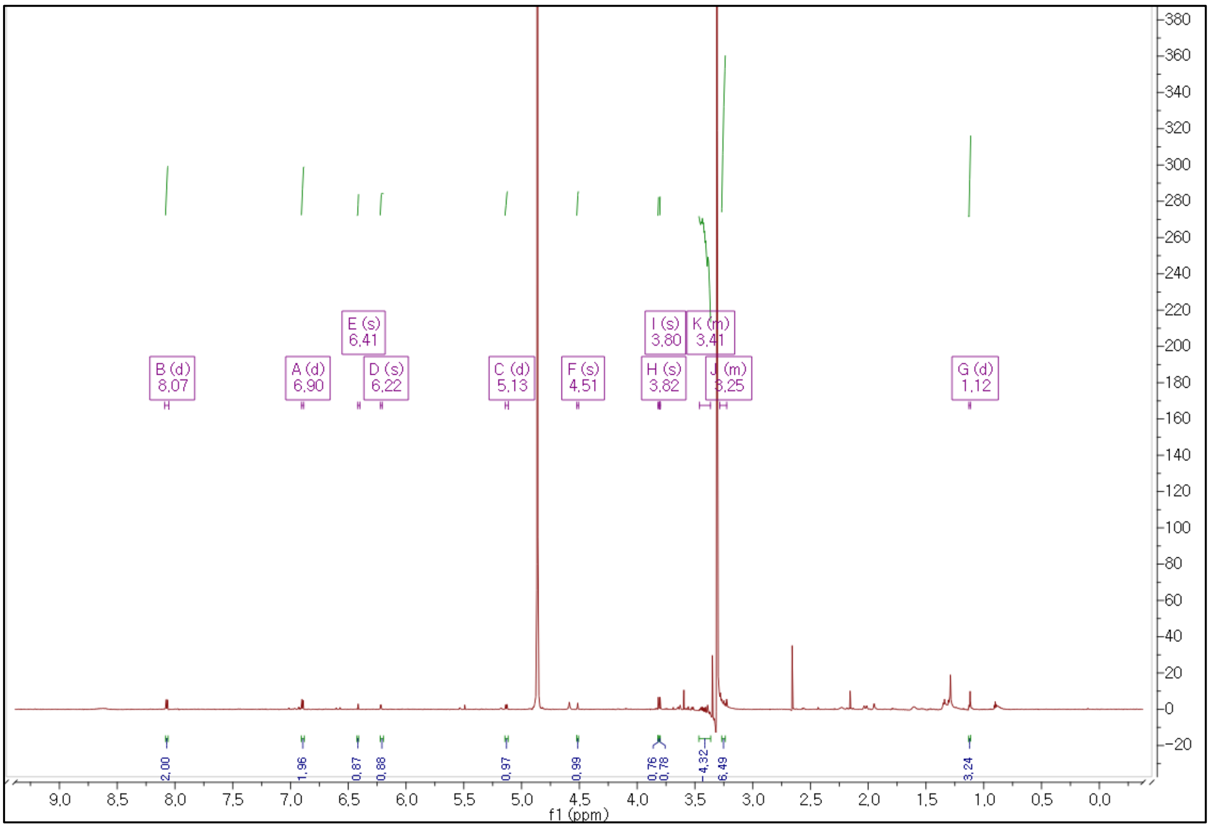

Figure S15. The ^1H NMR spectrum of compound **8** (CD_3OD, 850 MHz)

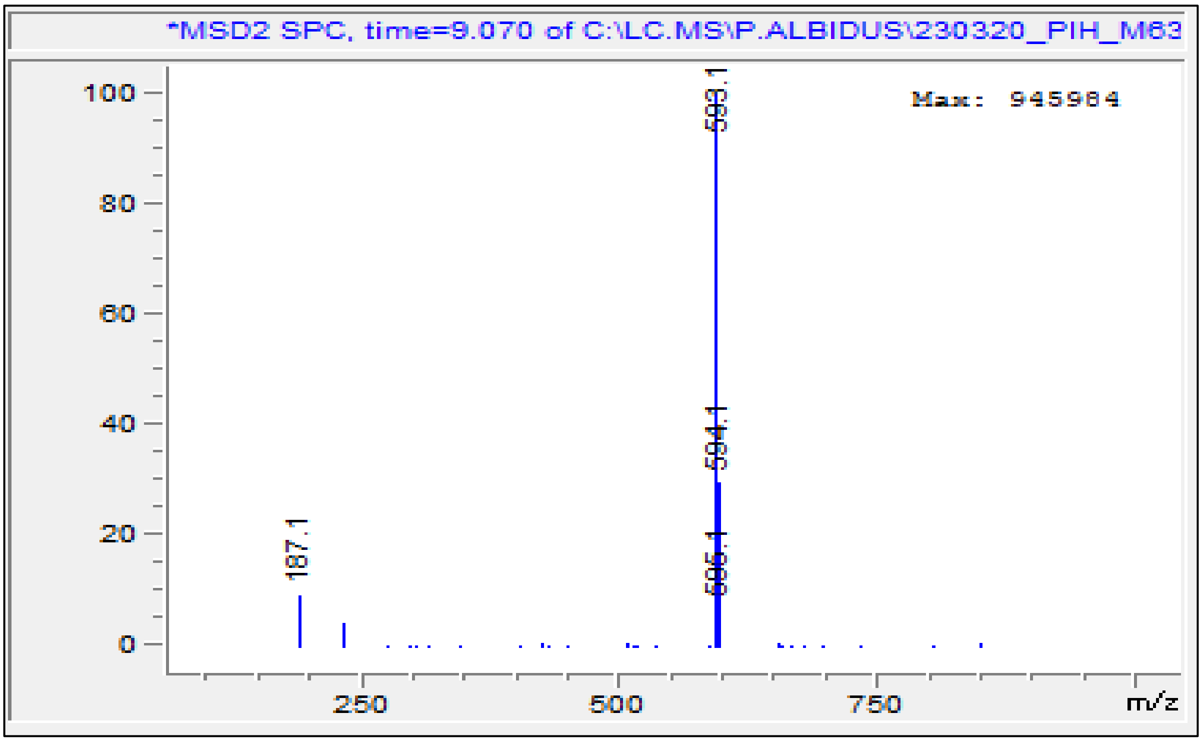

Figure S16. The ESIMS data of **8** (negative-ion mode)

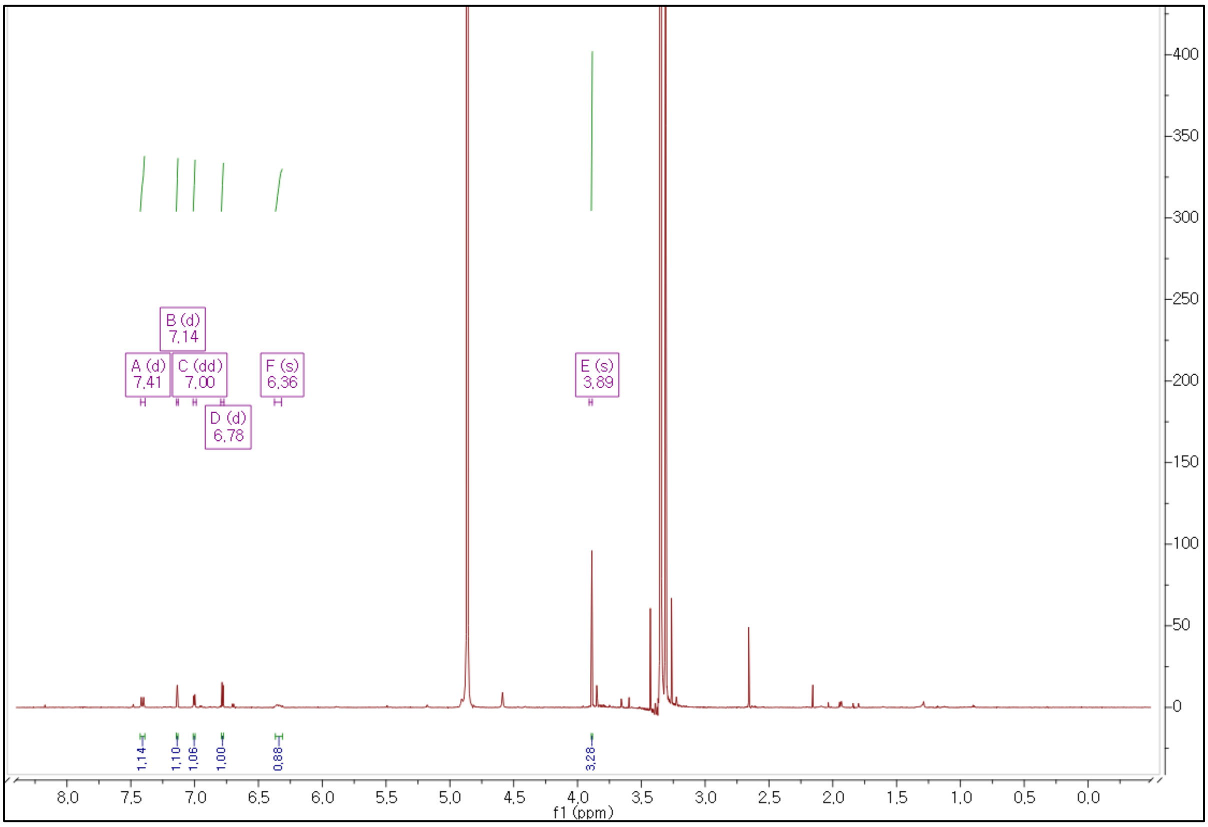

Figure S17. The ^{1}H NMR spectrum of compound **9** (CD_3OD, 850 MHz)

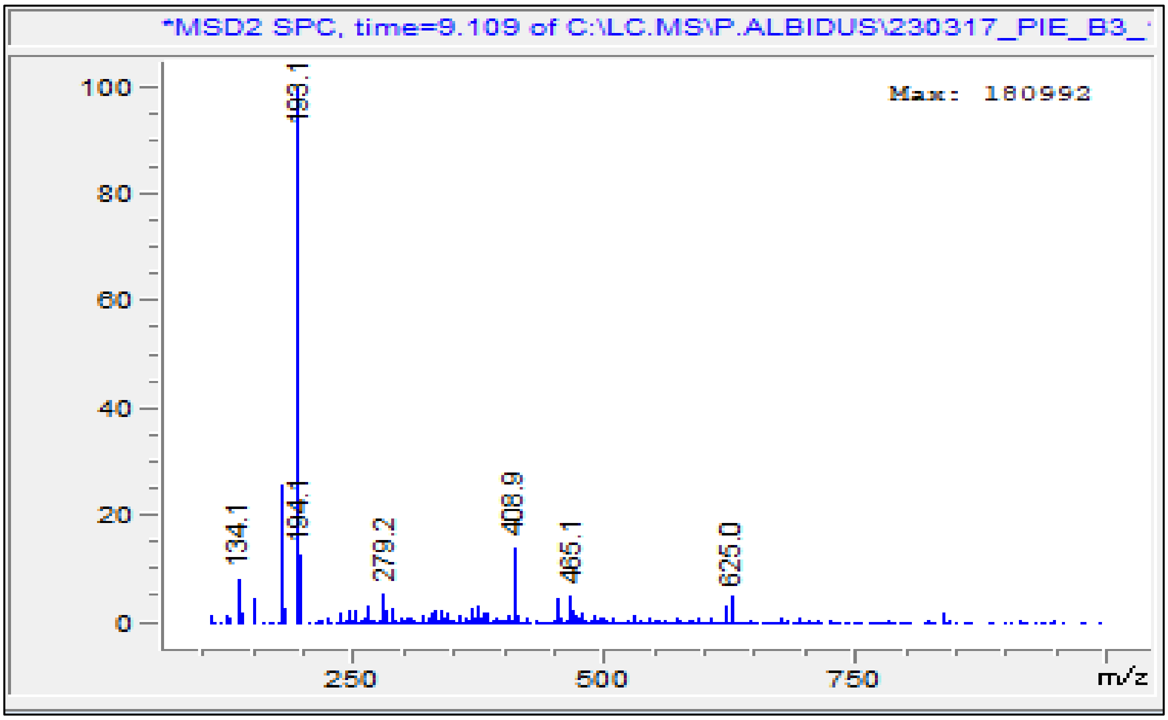

Figure S18. The ESIMS data of **9** (negative-ion mode)

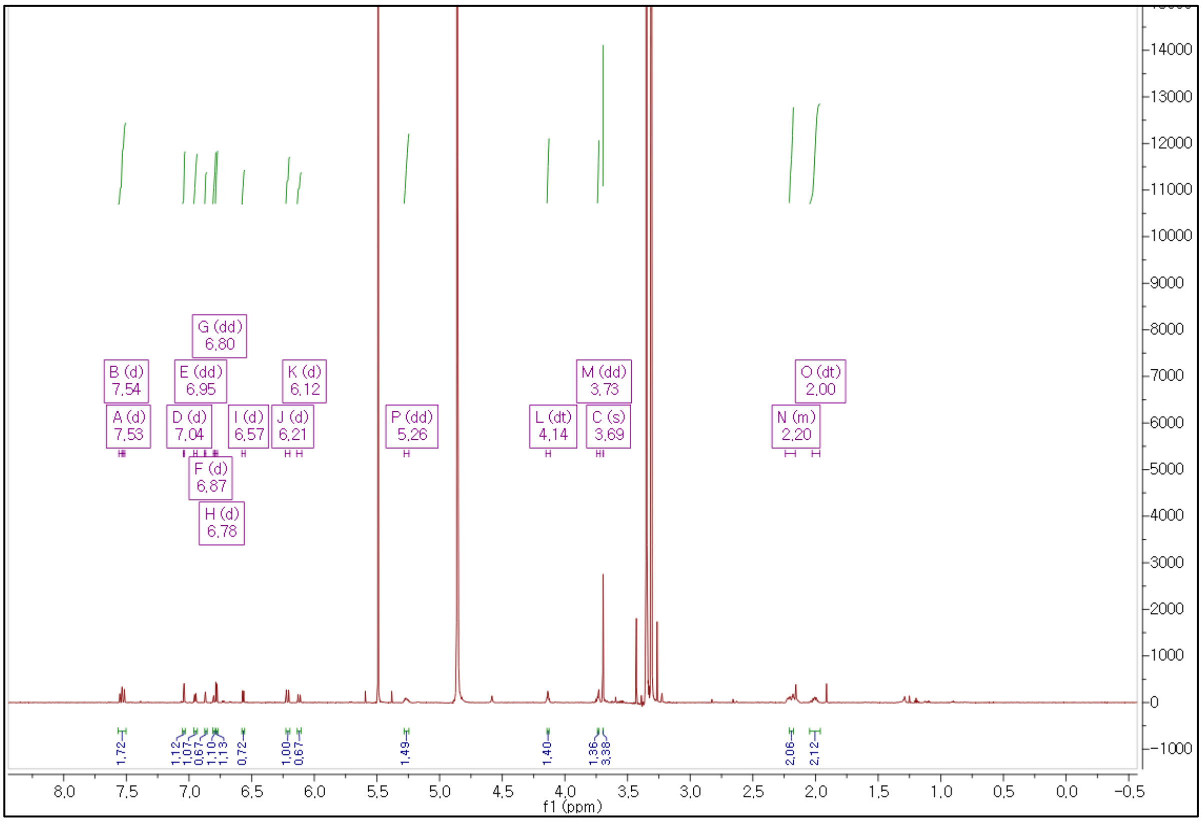

Figure S19. The ^{1}H NMR spectrum of compound **10** (CD_3OD, 850 MHz)

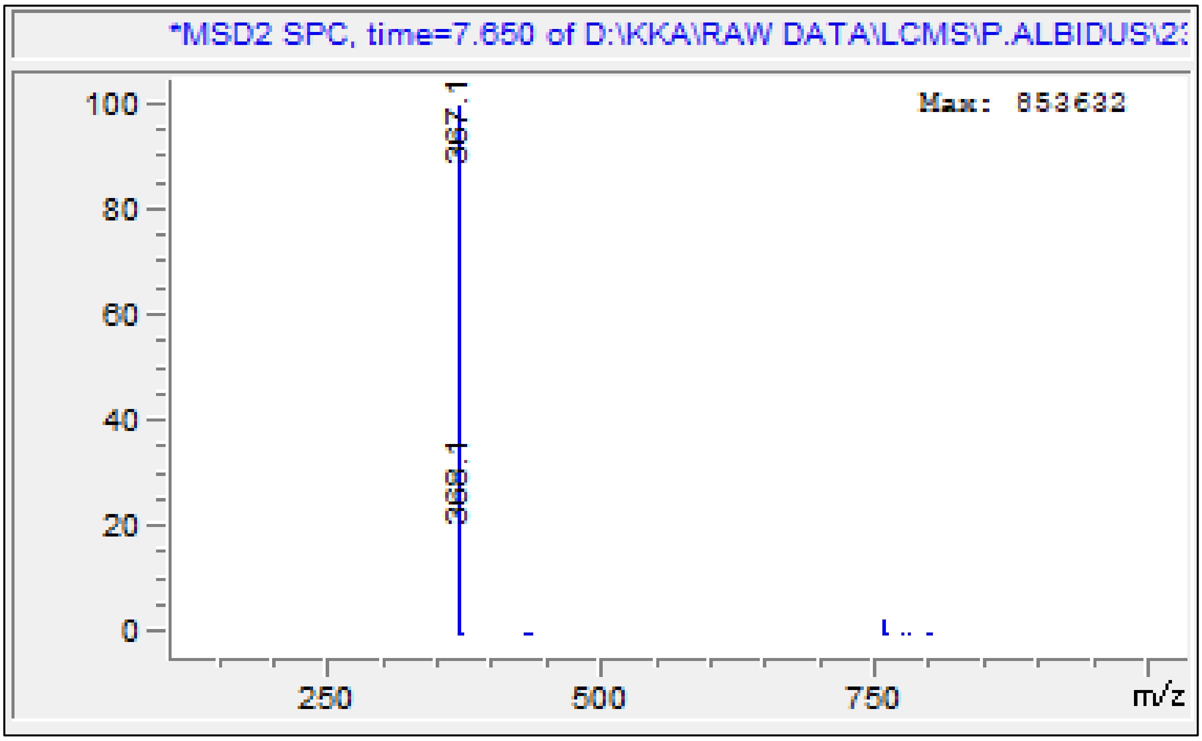

Figure S20. The ESIMS data of **10** (negative-ion mode)

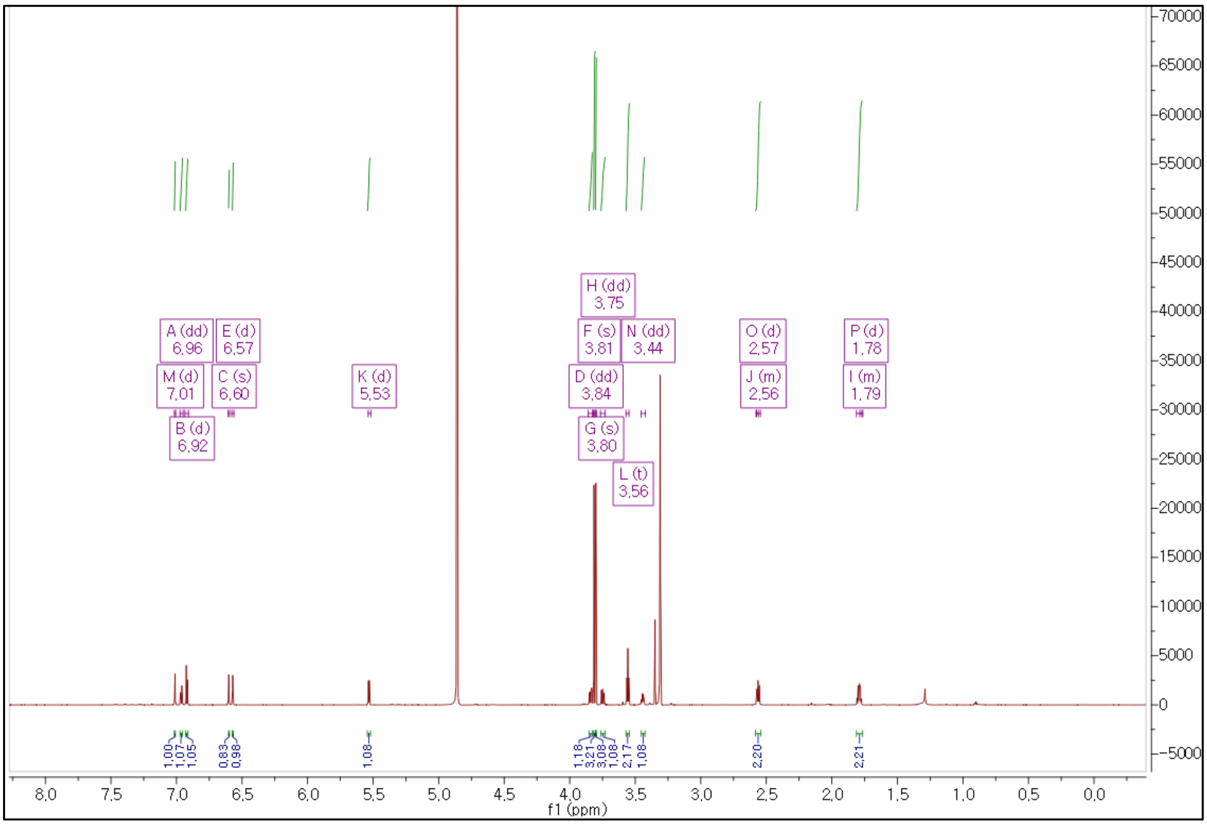

Figure S21. The ^{1}H NMR spectrum of compound **11** (CD_3OD, 850 MHz)

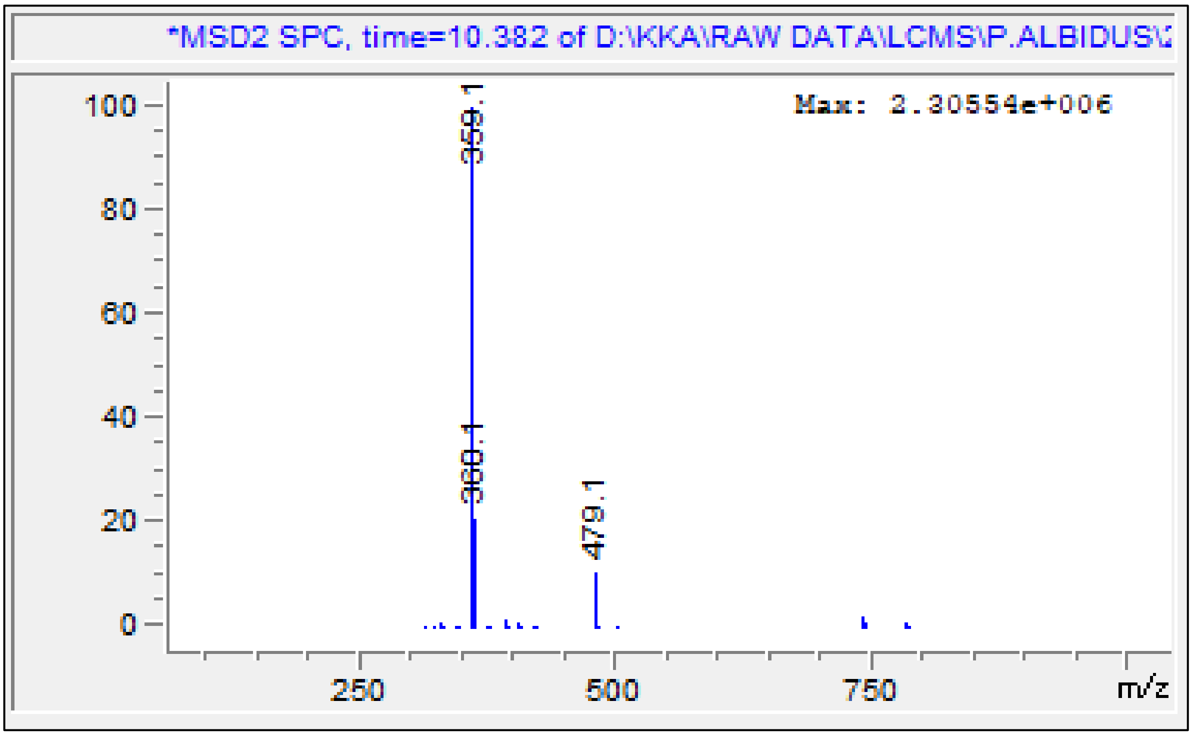

Figure S22. The ESIMS data of **11** (negative-ion mode)

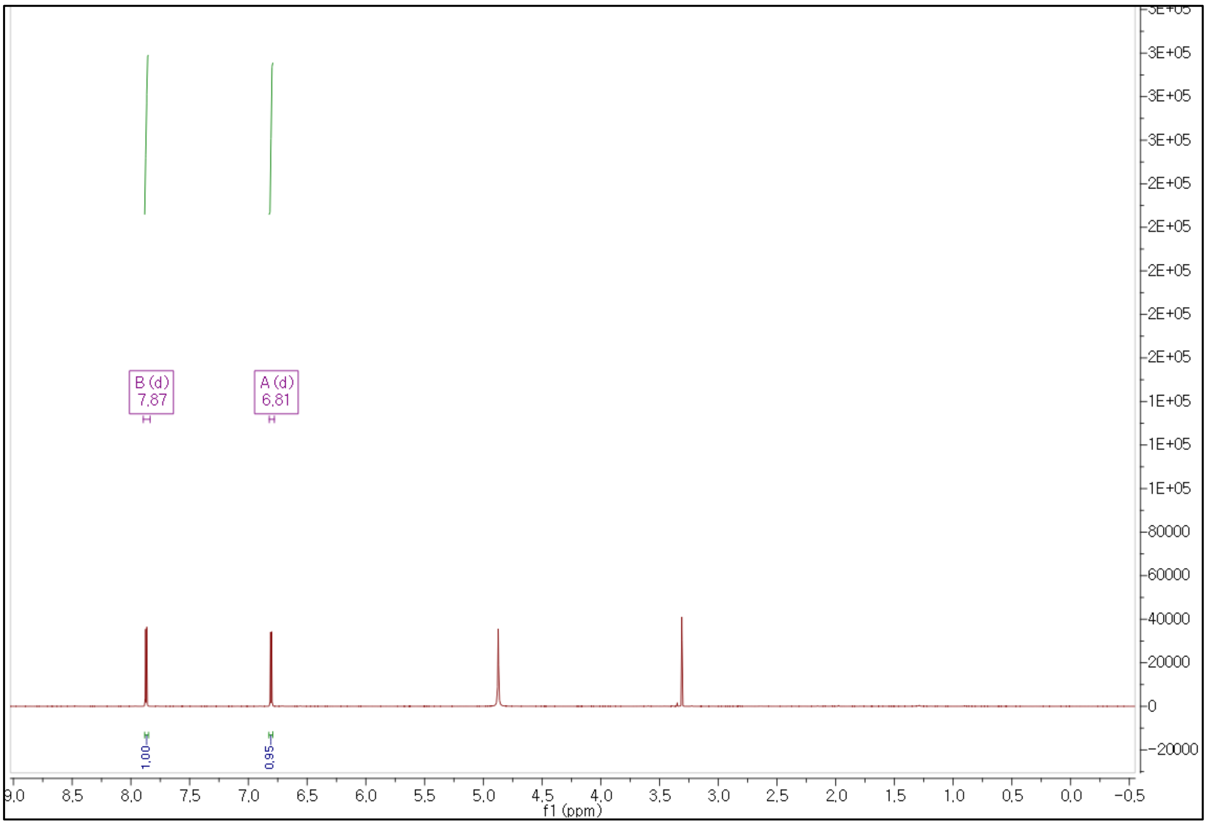

Figure S23. The ^{1}H NMR spectrum of compound **12** (CD_3OD, 850 MHz)

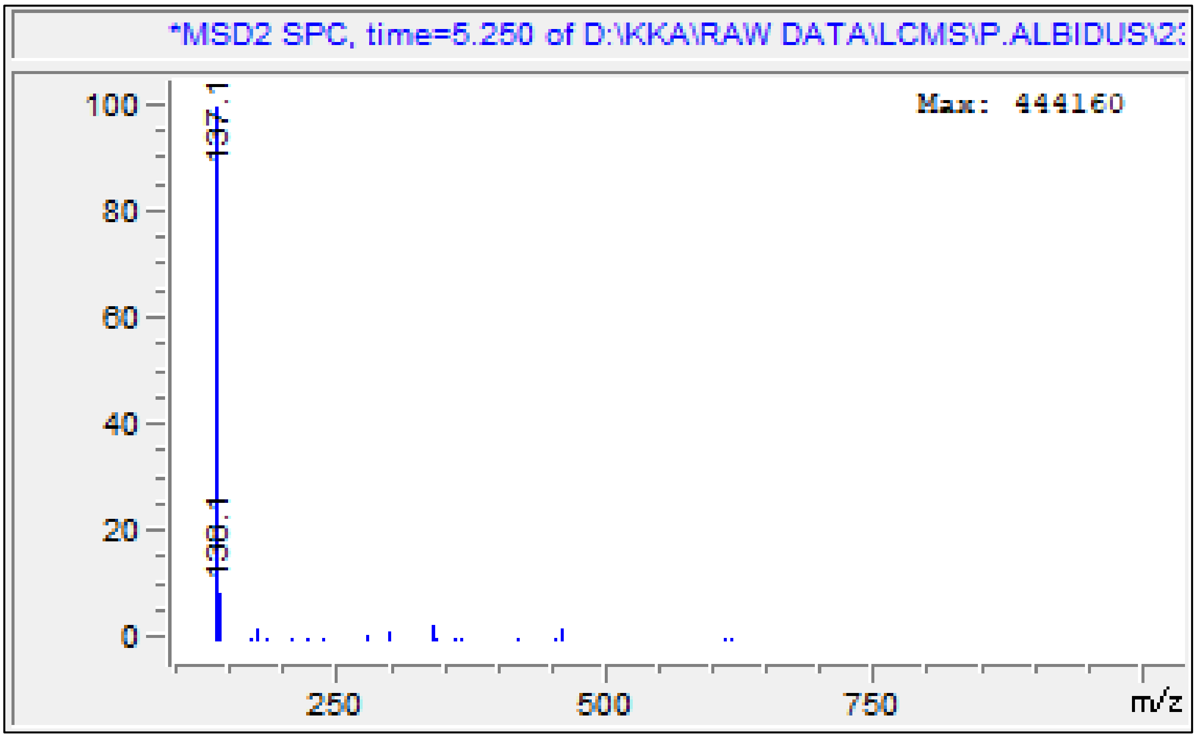

Figure S24. The ESIMS data of **12** (negative-ion mode)

Quantitative analysis for theophylline, theobromine, EGCG, and caffeine
Calibration Curves and Linearity

Quantitative analysis of the four target analytes (theobromine, theophylline, caffeine, and EGCG) was performed using LC–MS/MS in Selected Ion Monitoring (SIM) mode. Calibration curves were constructed by plotting peak area (Y) against nominal analyte concentration (X, mg/mL). The resulting linear regression equations and correlation coefficients (r^2) are summarized in Table 1. All analytes demonstrated excellent linearity across their respective calibration ranges with r^2 value exceeding 0.997, confirming the high reliability and reproducibility of method. The regression equations describe the quantitative relationship between analyte concentration and measured peak area, while the r^2 values reflected the consistency and accuracy of calibration across multiple concentration levels. Each calibration curve was generated using at least 5 concentration points to ensure robust, precise quantification (Table S1).

Table S1. Calibration curves of theophylline, theobromine, EGCG, and caffeine

Analyte	Calibration equation [1]	Correlation factor, r^2 [2]
Theophylline	Y = 4944.67X + 146018.36	0.9982
Theobromine	Y = 5359.06X + 828588.83	0.9975
EGCG	Y = 7,510,873.89X - 2119115.36	0.9976
Caffeine	Y = 9242.39X + 1572912.56	0.9973

[1] Y = peak area, X = concentration of standard (mg/mL).
[2] r^2 = Correlation coefficients for each analyte were determined from calibration curves constructed using at least five concentration levels.

Table S2. Concentrations of theophylline, theobromine, EGCG, and caffeine in *P. albidus* extracts and green tea extracts

Sample	Theophylline (%)	Theobromine (%)	EGCG (%)	Caffeine (%)
P. albidus 1	ND	ND	ND	ND
P. albidus 2	ND	ND	ND	ND
P. albidus 3	ND	ND	ND	ND
P. albidus 4	ND	ND	ND	ND
Green Tea (Control)	0.009	0.044	3.496	5.495

ND = not detected

Table S3. Primers and Sequences

Target Gene	Forward	Reverse
GAPDH	5′-AGAACGGGAAGCTTGTCATC-3′	5′-GGAGGCATTGCTGATGATCT-3′
IL-8	5′-CTCTGTGTGAAGGTGCAGTT-3′	5′-AAACTTCTCCACAACCCTCTG-3′
